# Acetate Supplementation Induces Growth Arrest of NG2/PDGFRα-Positive Oligodendroglioma-Derived Tumor-Initiating Cells

**DOI:** 10.1371/journal.pone.0080714

**Published:** 2013-11-20

**Authors:** Patrick M. Long, Scott W. Tighe, Heather E. Driscoll, John R. Moffett, Aryan M. A. Namboodiri, Mariano S. Viapiano, Sean E. Lawler, Diane M. Jaworski

**Affiliations:** 1 Department of Neurological Sciences, University of Vermont College of Medicine, Burlington, Vermont, United States of America; 2 Vermont Cancer Center, Burlington, Vermont, United States of America; 3 Vermont Genetics Network, Norwich University, Northfield, Vermont, United States of America; 4 Department of Anatomy, Physiology & Genetics, Uniformed Services University of the Health Sciences, Bethesda, Maryland, United States of America; 5 Department of Neurosurgery, Brigham and Women's Hospital, Boston, Massachusetts, United States of America; University of Portsmouth, School of Pharmacy & Biomedical Sciences, United Kingdom

## Abstract

Cancer is associated with globally hypoacetylated chromatin and considerable attention has recently been focused on epigenetic therapies. N-acetyl-L-aspartate (NAA), the primary storage form of acetate in the brain, and aspartoacylase (ASPA), the enzyme responsible for NAA catalysis to generate acetate and ultimately acetyl-Coenzyme A for histone acetylation, are reduced in oligodendroglioma. The short chain triglyceride glyceryl triacetate (GTA), which increases histone acetylation and inhibits histone deacetylase expression, has been safely used for acetate supplementation in Canavan disease, a leukodystrophy due to ASPA mutation. We demonstrate that GTA induces cytostatic G_0_ growth arrest of oligodendroglioma-derived cells *in vitro*, without affecting normal cells. Sodium acetate, at doses comparable to that generated by complete GTA catalysis, but not glycerol also promoted growth arrest, whereas long chain triglycerides promoted cell growth. To begin to elucidate its mechanism of action, the effects of GTA on ASPA and acetyl-CoA synthetase protein levels and differentiation of established human oligodendroglioma cells (HOG and Hs683) and primary tumor-derived oligodendroglioma cells that exhibit some features of cancer stem cells (grade II OG33 and grade III OG35) relative to an oligodendrocyte progenitor line (Oli-Neu) were examined. The nuclear localization of ASPA and acetyl-CoA synthetase-1 in untreated cells was regulated during the cell cycle. GTA-mediated growth arrest was not associated with apoptosis or differentiation, but increased expression of acetylated proteins. Thus, GTA-mediated acetate supplementation may provide a safe, novel epigenetic therapy to reduce the growth of oligodendroglioma cells without affecting normal neural stem or oligodendrocyte progenitor cell proliferation or differentiation.

## Introduction

 Glioma, the most common primary brain tumor of the adult central nervous system, is associated with a poor prognosis. Standard therapy of maximal surgical resection followed by concurrent radiotherapy and temozolomide (Temodar^®^) chemotherapy [1] has increased median survival for patients with glioblastoma (GBM, WHO grade IV astrocytoma) to ~14 months. Perhaps due to the decreased incidence of oligodendroglial tumors (i.e., 0.27 per 100,000 persons vs. 3.19 per 100,000 persons for GBM) (CBTRUS 2005-2009 report) [2], less attention has been focused on developing oligodendroglioma therapies. Inasmuch as the median survival for patients with anaplastic oligodendroglioma without loss of heterozygosity (LOH) of 1p and 19q chromosome arms is comparable to that of GBM (i.e., 1.2 years) [3], studies focusing on novel oligodendroglioma therapeutic strategies are warranted.

Despite multimodal therapeutic approaches, tumor recurrence is nearly inevitable in high-grade glioma patients. Although the precise mechanism underlying recurrence is unknown, the post-surgical persistence of radiation and chemotherapy resistant tumorigenic cells with stem cell characteristics (i.e., self-renewal and multi-lineage differentiation) has been postulated as a contributing factor [4]. Thus, therapeutic approaches that selectively target these glioma stem cells (GSCs), while sparing normal neural stem cells (NSCs) and oligodendrocyte progenitor cells (OPCs), the most abundant cycling population in the adult brain [5], are of considerable interest.

Acetate supplementation may prove to be a novel, efficacious glioma therapeutic avenue since it acts at the intersection of epigenetics and metabolism, two hallmarks of aggressive tumor growth. Promoter CpG hypermethylation is coupled to histone hypoacetylation and silencing of tumor-suppressor genes, with increased histone hypoacetylation correlated with poorer clinical outcomes [6]. Under normal nutrient conditions, most nuclear acetyl-Coenzyme A (acetyl-CoA) needed for histone acetylation is derived from glucose via conversion of mitochondrial-derived citrate by ATP-citrate lyase [7]. However, mitochondrial citrate is exported to the cytosol in highly proliferative, glycolytically converted tumor cells to support lipid synthesis and biomass accumulation required for proliferation [8]. Thus, under glycolytic conditions, an alternative nucleocytosolic acetyl-CoA synthetic pathway becomes relevant.

N-acetyl-L-aspartate (NAA) is the most concentrated source of acetate in the human brain (~12.1 ± 1.5 mM) [9]. The lower NAA level in white matter (9.5 ± 1.0 mM) than grey matter (14.3 ± 1.1 mM) [10] is likely due to a greater NAA utilization rate by oligodendrocytes for myelin lipid biosynthesis and protein acetylation reactions. Aspartoacylase (ASPA) catalyzes the breakdown of NAA, its only known substrate [11], to L-aspartate, for use in protein synthesis and the Krebs cycle, and acetate. Although the precise metabolic fate of NAA-derived acetate is uncertain, evidence suggests that two enzymes are capable of converting free acetate to acetyl-CoA: cytosolic/nuclear acetyl-CoA synthetase-1 (AceCS1) for lipid biosynthesis and histone/protein acetylation [12] and mitochondrial AceCS2 for ATP production [13]. Both NAA and ASPA protein levels are decreased in glioma [14,15]. Thus, decreased NAA-derived acetate bioavailability could promote transcriptional repression via reduced histone acetylation and exacerbate the Warburg effect (i.e., aerobic glycolysis) [16,17]. NAA supplementation using mono-methyl NAA, which crosses membranes, is a possible therapeutic approach. However, NAA is rapidly taken up by astrocytes and excreted to the circulation [14]. Moreover, NAA catalysis requires ASPA, which is decreased in glioma. Most importantly, NAA and its derivative metabolite N-acetylaspartylglutamate (NAAG) promote GSC proliferation *in vitro* [18]. Thus, an alternative acetate source is required.

Triacetin (glyceryl triacetate, GTA) is ideal for therapeutic acetate supplementation. Unlike free acetate, GTA is hydrophobic and freely crosses the blood-brain barrier and plasma membranes and is hydrolyzed by non-specific lipases and esterases in all cell types to liberate glycerol and acetate. Glycerol can either participate in *de novo* triglyceride synthesis or be used for glycolysis after conversion to glyceraldehyde-3-phosphate, while the acetate generates acetyl-CoA [19]. We demonstrate that GTA induces cytostatic growth arrest of primary tumor-derived oligodendroglioma cells (grade II OG33 and grade III OG35) that possess some features (i.e., self-renewal and the formation of aggressive orthotopic tumors) of OG cells more than established human oligodendroglioma cells (HOG and Hs683), but has little to no effect on normal cells (i.e., NSCs and a murine OPC line, Oli-Neu). Interestingly, GTA was more effective than sodium acetate. The spatial localization of ASPA and AceCS1 in the nucleus was regulated during the cell cycle. GTA-mediated acetate supplementation did not induce anti-proliferative effects via the promotion of apoptosis or differentiation, but increased acetylation of several proteins involved in cell cycle regulation, suggesting an epigenetic mechanism of action.

## Materials and Methods

### Ethics Statement

Research involving human tumor tissue and tumor-derived cells was conducted under approved institutional protocols. A human oligodendroglioma tumor, used as a positive control for RNA profiling, was obtained under University of Vermont Institutional Review Board (IRB) protocol # CHRMS: M09-060 (DMJ). Although the tissue would normally be discarded and not require written consent to be obtained under IRB guidelines, the Vermont Cancer Center Protocol Review Committee (PRC), which oversees cancer related, research required written consent. Thus, the tumor sample was obtained under PRC protocol # VCC-0808 (DMJ). The oligodendroglioma-derived cells, OG33 and OG35, and the GBM-derived cells (GBM2, 8, 9, 12, 34, 44) were established at The Ohio State University (OSU) under IRB# 2005C0075 and subsequently transferred to Brigham and Women's Hospital (BWH) protocol IRB# 2012P002661/1 (MSV). Similar to UVM IRB policy, both OSU and BWH IRBs consider discarded tissue exempt from consent. Thus, consent was not obtained.

### Cell Culture

Established oligodendroglioma cell lines, HOG (courtesy of Dr. Glyn Dawson, University of Chicago Department of Pediatrics) and Hs683 (HTB-138; American Type Culture Collection; Manassas, VA), were maintained in Dulbecco’s Modified Eagle Medium (DMEM; Mediatech; Herndon, VA) supplemented with 5% or 10% fetal bovine serum (FBS; Hyclone; Logan, UT), respectively, on untreated cell culture dishes. HOG cells were derived from an oligodendroglioma [20] while the Hs683 line was derived from an explant culture of a glioma taken from the left temporal lobe of a 76-year-old male [21]. The Oli-Neu cell line, derived from murine OPCs immortalized by stable constitutive expression of the ErbB2 receptor [22], was grown on poly-L-lysine (PLL; 10 μg/ml) coated dishes in SATO growth medium (GM, DMEM with 0.1 mg/ml apotransferrin, 0.01 mg/ml insulin, 400 nM triiodothyronine, 2 mM glutamine, 200 nM progesterone, 100 μM putrescine, 220 nM sodium selenite, 500 nM thyroxine, 1% horse serum, and 25 µg/ml G418) [23]. Primary tumor-derived cells with self-renewal and tumorigenic capacity were isolated from surgical specimens using previously described methodology [24]. OG33 cells were derived from a WHO grade II oligodendroglioma taken from the left frontal lobe of a 45 year old male while OG35 cells were derived from a grade III oligodendroglioma taken from the right frontal lobe of a 34 year old female. All GBM-derived cells were isolated from frontal lobe tumors: GBM2 from a 47-year-old male, GBM8 from a 70-year-old female, GBM34 from a 78-year-old female, GBM44 from a 44-year-old male, while GBM12 was derived from a recurrent tumor in a 64-year-old female from which GBM9 was originally established. The OG and GBM primary tumor-derived cells were kindly provided by Dr. Antonio Chiocca (Brigham and Women's Hospital Department of Neurosurgery). The OG and GBM primary tumor-derived cells were maintained as free-floating spheres in stem cell medium (SCM) consisting of DMEM/F12, 1X B27 supplement (Life Technologies; Grand Island, NY), 20 ng/ml EGF and 20 ng/ml bFGF (PeproTech; Rocky Hill, NJ) on non-adhesive plastic (Falcon petri dish). All medium contained 50 U/ml penicillin and 50 μg/ml streptomycin and was replenished every 48 hours.

Oli-Neu cell differentiation was induced by incubation in modified SATO medium (DMEM supplemented with 100 µg/ml apotransferrin, 5 µg/ml insulin, 60 nM triiodothyronine, 30 nM sodium selenite, 100 µM putrescine, 1% horse serum, and 25 µg/ml G418) [25]. OG33 and OG35 differentiation was induced using DMEM with 10% FBS (differentiation medium, DM), adipogenic (α-MEM containing 10% FBS, 0.5 μM hydrocortisone, 0.5 mM isobutylmethylxanthine, and 60 μM indomethacin) or osteogenic (α-MEM containing 10% FBS, 1 nM dexamethasone, 0.2 mM ascorbic acid, and 10 mM β-glycerol phosphate) medium as described [26-28]. After 3 weeks in osteogenic medium, cells were fixed with 2% paraformaldehyde for 20 min, washed twice for 10 min each with phosphate buffered saline, stained with 0.5% Alizarin Red S (pH 4.1; Sigma) for 20 min, and washed three times for 5 min each with phosphate buffered saline. For pharmacological induction of differentiation, OG33 and OG35 cells were plated in SCM or DM in the absence or presence of dibutyryl cAMP (1 mM in water, Sigma), forskolin (10 μM in ethanol, Sigma), the MEK1/2 inhibitor PD035901 (1 μM in dimethylsulfoxide [DMSO], Tocris Bioscience/R & D Systems; Minneapolis, MN), the ErbB2 inhibitor PD174285 (1 μM in DMSO, Santa Cruz Biotechnology), the PI3K inhibitor LY294002 (at 5 μM and 10 μM in DMSO, Tocris), the mTOR inhibitor rapamycin (at 10 nM and 20 nM in ethanol, Sigma) or 0.2% DMSO as a control (final DMSO concentration of 0.05% for PD174285, 0.1% for PD035901 and LY294002, and 0.2% for PD035901/LY294002/rapamycin treatment). Medium was replenished every 48 hours and cells were harvested after 6 days. 

For western blot analysis, cells were plated at a density of 10,000 cells per cm^2^ per 6-cm dish. For immunocytochemistry, cells were plated at a density of 20,000 cells per well of 24-well plate. The established cell lines were cultured overnight in GM, then cultured with fresh GM in the absence or presence of 0.25% GTA (Sigma) and cells were harvested after 2, 4, and 6 days *in vitro* (i.e., 1, 3, 5 days of treatment). The GTA concentration (0.25%, ~12 mM) was selected based on a dose response and represents the lowest concentration that induced growth arrest in both SCM and DM [15]. OG33 and OG35 cells were cultured in SCM as floating spheres or as adherent monolayers on PLL treated plates overnight, then cultured with fresh SCM in the absence or presence of 0.25% GTA for an additional 24 hours and harvested. OG33 and OG35 cells were induced toward an oligodendroglial cell fate by culturing in DM in the absence of GTA for 24 hours, then for an additional 1, 3, or 5 days in the absence or presence of 0.25% GTA. To detect proteins acetylated consequent to GTA treatment, cells were incubated with 0.25% GTA and cells were harvested 6, 12, or 24 hours later. Because protein acetylation is transient, cells were also treated with GTA in the absence or presence of the histone deacetylase inhibitor (HDACi) suberoylanilide hydroxamic acid (SAHA, Vorinostat) (1 μM, Sigma) and cells were harvested 24 hours later. In all cases, medium was replenished every 48 hours. 

### Short tandem repeat (STR) profiling

Cells were validated at the Vermont Cancer Center DNA Analysis Facility by STR DNA fingerprinting [29] using the CELL IDTM System according to manufacturer's instructions (#G9500, Promega; Madison, WI).  The STR profiles were compared to known ATCC fingerprints (www.ATCC.org) and to the Cell Line Integrated Molecular Authentication (CLIMA) database version 0.1.200808 (http://bioinformatics.istge.it/clima/). 

### Reverse transcription PCR (rtPCR) profiling

For proneural and mesenchymal antigenic profiling, cells (2.5 x 10^6^) were cultured in SCM for 4 days and total RNA extracted with 1 ml Stat-60 (Tel-Test B; Friendswood, TX) according to manufacturer's instructions. RNA (1 μg) was reverse transcribed using Super Script II reverse transcriptase (Life Technologies; Grand Island, NY) with random hexamers. A human oligodendroglioma tumor served as a positive control. The cDNA (1 μl) was amplified using HotStarTaq master mix (Qiagen; Valencia, CA) in a 20 μl reaction volume. After a 10 min 98°C activation step, cycling parameters of 95°C for 30 sec, 58°C for 30 sec and 72°C for 30 sec were repeated 32 times followed by a 1 min final extension at 72°C. PCR products (10 μl) were resolved via agarose gel electrophoresis and visualized with ethidium bromide using a Chemidoc gel imaging system (Bio-Rad Laboratories; Hercules, CA).

DNA sequencing was undertaken to determine whether the oligodendroglioma cells harbored known IDH1 and IDH2 mutations [30,31]. PCR primers corresponding to the genomic regions of exon 4 containing codon R132 of IDH1 and exon 4 containing codon R172 of IDH2 were used to amplify 100 ng genomic DNA using HotStarTaq master mix. After 98°C heat activation for 30 sec, cycling parameters of 98°C for 5 sec, 60°C for 5 sec and 72°C for 20 sec were repeated 30 times followed by a 1 min final extension at 72°C, amplicons were electrophoresed and purified from agarose gels, and DNA sequencing performed using the ABI PRISM 3100 Avant Genetic Analyzer at the Vermont Cancer Center DNA Analysis Facility. Sequence traces were examined using FinchTV (Geospiza; Seattle, WA) and compared to IDH1 (Accession AF020038) or IDH2 (Accession X69433). 

To determine whether the novel 26 kDa ASPA isoform could arise from the most common ASPA mutation (i.e., Y231X), genomic DNA was analyzed as described above for IDH1/2. Sequence traces were compared to ASPA Accession NM_000049.2. All primer sequences are detailed in Methods S1. 

### Whole genome cytogenetic analysis

DNA mapping was performed using the GeneChip® Human Mapping 250K Nsp Array (Affymetrix; Santa Clara, CA). Genomic DNA (250 ng) was processed according to the manufacturer's protocol. Briefly, genomic DNA was cut with Nsp restriction enzyme followed by ligation with Nsp adaptors that included a known sequence used for amplification by PCR. Thirty cycles of PCR was used to amplify the entire genome followed by cleaning and fragmentation using DNase I. Fragmented DNA was end-labeled with biotin using a standard terminal deoxynucleotidyl transferase reaction and confirmed with a gel shift assay. Samples were hybridized to the Affymetrix 250K Nsp Array for 16 hours at 49°C followed by a double streptavidin-phycoerytherin staining and scanned on a GS3000-7G scanner. All CEL files were corrected for probe GC content and fragment length. CEL files produced by Affymetrix GeneChip® Operating Software with a QC call rate of 92.5 or greater were analyzed for gross chromosomal copy number alterations using the Affymetrix Genotyping Console 4.1 and Integrated Genome Browser and compared against gender matched normal samples from the International HapMap project Database (NCBI). Forty single nucleotide polymorphism (SNP) marker resolution was used to eliminate false positives. Principal component analysis (PCA) plots were generated using raw probe intensities and copy number was estimated by comparing raw probe intensities to Partek’s distributed baseline from the International HapMap Project (NCBI). Genomic regions with shared copy number variation were determined using the Hidden Markov Model algorithm implemented in Partek set to detect copy number (CN) states of 0.1, 1, 3, 4, 5 (a CN state of 2 was ignored), with the minimum number of probe sets contained in a region for it to be considered set to 3. Karyotype plots were used to visualize genomic regions shared across samples.

### Growth assessment

Cell cycle kinetics were assess 24 hours after treatment with 0.25% GTA, 0.25% glycerol, 0.25% triglyceride (canola oil), 36 mM sodium acetate (equivalent acetate to 0.25% GTA) or 12 mM sodium acetate (since 3 moles of acetate are derived per mole of GTA). Cell cycle profiles were visualized by propidium iodide (PI) staining as described [32] with minor modifications (10^6^ cells/ml were incubated in low-salt PI solution at 37°C for 20 minutes, then an equal volume of high-salt PI solution was added and incubated at 4°C for 4 hours). Cell cycle profiles were recorded using the BD LSR II Flow Cytometer (BD Biosciences; San Jose, CA) and analyzed using FACS Diva 7.0 software.

Growth dynamics were assessed using unbiased trypan blue exclusion based cytometry. Cells were plated (at 10,000 cells per well of a 24-well plate) directly in the absence or presence of 0.25% GTA, 0.25% glycerol, 0.25% triglyceride, 36 mM sodium acetate, 12 mM sodium acetate or 36 mM sodium acetate plus 0.25% glycerol. Medium was replenished every 48 hours. After 5 days of treatment, medium was collected, centrifuged briefly to remove cellular debris, and pH measured using a standard pH meter. Because GTA resulted in significant medium acidification in DM between days 3 and 5, medium was acidified to pH 6.5 with hydrochloric acid (glacial acetic acid was not used since it could serve as an acetate source), sterile filtered, and added to cultures at day 3. After 1, 3, and 5 days of treatment, cells were typsinized, collected via centrifugation, and counted according to the manufacturer’s instructions (Countess Automated Cell Counter; Invitrogen). 

### Protein Analysis

SDS-PAGE (25 μg protein from whole cell lysates) and western blotting were performed as described [33]. OG33 cells were treated with physiological levels of NAAG (10 μM) for 4 days and subcellular cytosolic and nuclear fractions isolated as described [18]. Immunocomplexes were visualized by enhanced chemiluminescence (PerkinElmer Life Sciences; Boston, MA) with antibodies titred so that x-ray film (Kodak Biomax MR) exposure times would be comparable and within the linear range. Films were scanned using identical settings with an Epson Expression 800 flatbed scanner possessing a transparency adapter and imported into a single file using Photoshop CS version 8.0 (Adobe Systems; San Jose, CA). Semi-quantitative densitometry was performed using Quantity One software (Bio-Rad) by overlaying each band with an identical sized rectangle to encompass the entire band without overlapping adjacent bands, volume analysis (pixel count) performed, and relative protein levels determined by dividing the raw pixel count of the protein of interest by the raw pixel count of the normalizing protein using Excel software version 14.3.6 (Microsoft; Redmond, WA). Antibodies used in the study are detailed in the Methods S1.

Immunocytochemistry was performed as described [34]. All cytological images were acquired with identical exposure settings using a SPOT RT digital camera (Diagnostic Instruments; Sterling Heights, MI). Antibodies used in the study are detailed in the Methods S1.

### Statistical Analysis

Statistical analyses were performed using a minimum of three independent cultures. Data are expressed as means ± standard error of the mean. Significant differences were determined by either one-way or two-way ANOVA and Bonferroni multiple comparison tests using Prism software version 5.0 (GraphPad; San Diego, CA). p < 0.05 was considered statistically significant. 

## Results

The term oligodendroglioma was created based on the observation that these tumors possess cells with morphological similarities to oligodendrocytes [35], yet only recently has a convincing link between oligodendrocyte lineage cells and oligodendrogliomas been established. Evidence is mounting to support adult OPCs as a glioma cell of origin [36-39]. Hence, this study was undertaken to investigate the growth regulatory effects of acetate supplementation on ASPA and AceCS1 protein levels in established oligodendroglioma cells (HOG and Hs683) relative to primary oligodendroglioma-derived cells that exhibit self-renewal and tumorigenicity (grade II OG33 and grade III OG35) [15].

To determine whether differences in GTA responsiveness were correlated with chromosomal alterations, all human cell lines used in this study were subjected to in-depth DNA analysis. The STR DNA fingerprint for the commercially available Hs683 cell line matched its reported DNA fingerprint, while the profiles of the HOG, OG33, and OG35 cells did not match known DNA fingerprints (Table S1). OG33 and OG35 cells displayed different STR profiles indicating their distinct origin.

**Figure 1:**
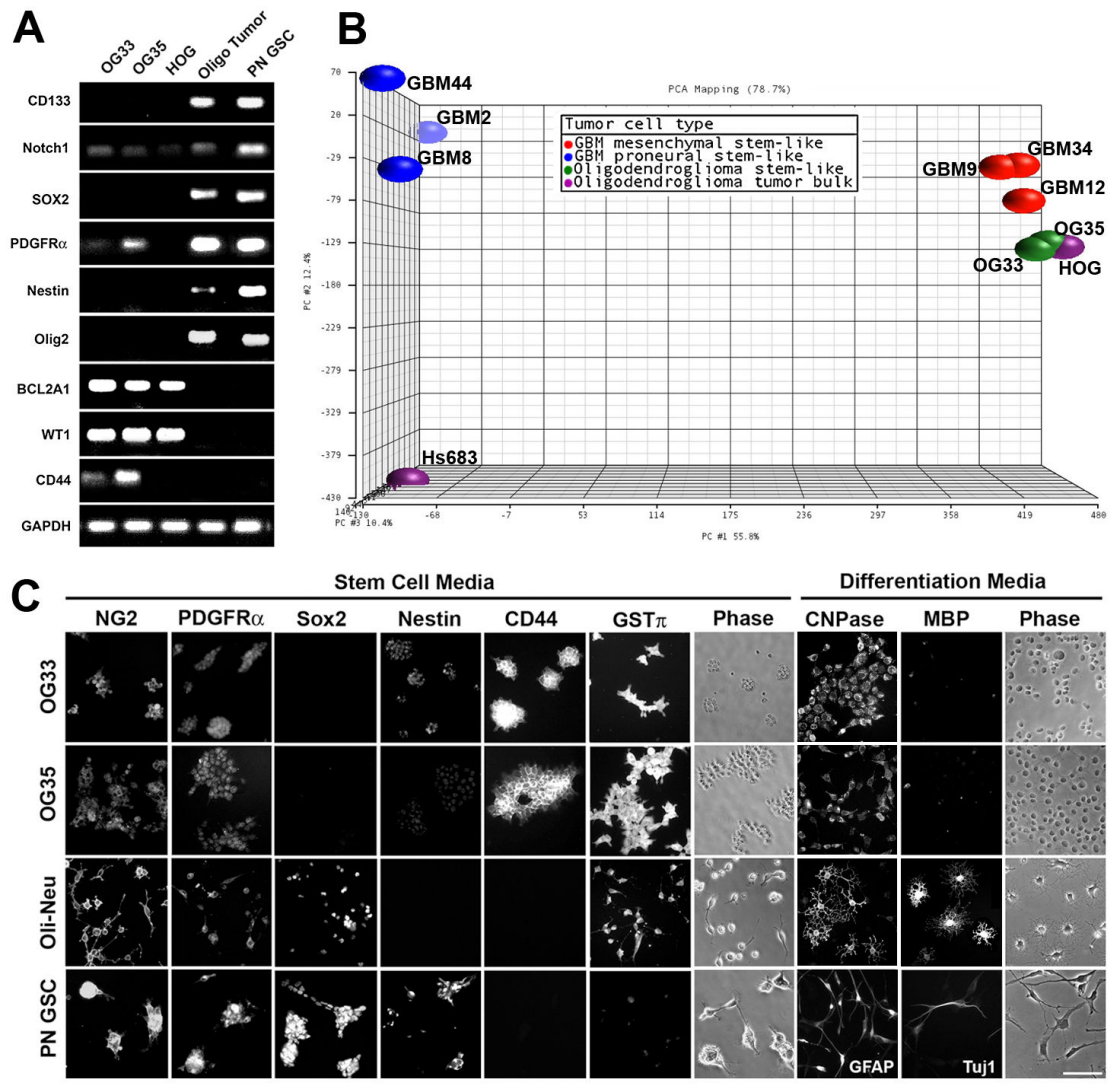
OG33 and OG35 cells exhibit a mesenchymal profile. A) PCR of OG33 and OG35 cells (grade II and grade III, respectively, primary oligodendroglioma-derived cells that exhibit self-renewal and tumorigenicity) maintained as floating spheres in stem cell medium for 4 days. A grade III oligodendroglioma tumor (Oligo tumor) and proneural (PN) GBM GSCs served as controls. Glyceraldehyde-3-phosphate dehydrogenase (GAPDH) was used as a loading control. OG cells expressed the OPC marker PDGFRα and Notch1, but lacked the neural stem cell markers CD133, SOX2, and Olig2. In contrast, they abundantly expressed the mesenchymal markers CD44, BCL2A1, Wilms Tumor 1 (WT1). B) Principal component analysis (PCA) of SNP raw intensity data from GeneChip® Human Mapping 250K Nsp Arrays. OG cells share similar gene amplifications/deletions to the established HOG oligodendroglioma cell line. Furthermore, the OG cells were more similar to mesenchymal GBM GSCs (GBM12, GBM9, and GBM34) than proneural GSCs (GBM44, GBM8, and GBM2). The Hs683 cell line, which was derived from a GBM tumor but shares features of oligodendroglioma tumors, failed to cluster with either tumor type. C) Immunocytochemistry after 3 days growth in stem cell medium (adherent on PLL) or differentiation medium revealed that the OG cells express a transition mesenchymal profile. In stem cell medium, cells expressed OPC markers (NG2 and PDGFRα), although less abundantly than Oli-Neu OPCs. In contrast, they abundantly expressed the mesenchymal markers CD44 and glutathione S-transferase π (GSTπ). In differentiation medium, OG33 and OG35 cells expressed lower levels of CNPase than Oli-Neu cells, and, unlike Oli-Neu cells, the OG cells failed to express myelin basic protein (MBP), a well-accepted marker of mature oligodendrocytes. Oli-Neu and PN GBM GSCs served as controls. Scale bar = 100 µm.

Inasmuch as a proneural signature has been proposed for oligodendrogliomas [40,41], OG33, OG35, and HOG cells were subjected to phenotype profiling by rtPCR using well-accepted proneural and mesenchymal markers [42,43] (Figure 1A). OG cells did not express the proneural markers CD133, Sox2 or Olig2, but abundantly expressed the mesenchymal markers CD44, BCL2A1, and Wilms Tumor 1. Most importantly, the OG cells expressed platelet-derived growth factor receptor-alpha (PDGFRα), a well-accepted OPC marker (previously referred to as O2A progenitors) [44]; thus, supporting their oligodendroglial origin.

DNA mapping for chromosomal copy variation using GeneChip® 250K Nsp arrays revealed similar amplifications and deletions in HOG, OG33, and OG35 cells (Figure S1). This analysis confirmed the surgical pathology report of the allelic loss of 1p36/19q13, the most common genetic change in oligodendroglial neoplasms [3]. Principal component analysis (PCA) with the SNP raw intensity data grouped OG33, OG35, and HOG cells together, indicating similar copy variations, and that these cytogenetic alterations were distinct from mesenchymal or proneural GBM-derived GSCs (Figure 1B). Interestingly, the chromosomal alterations in oligodendroglioma-derived cells were more similar to mesenchymal GBM GSCs than proneural GSCs. Although Hs683 cells were established from a GBM, they display typical oligodendroglioma features, including 1p/19 co-deletion, temozolomide sensitivity, abundant integrin β4 and low integrin β1 expression, and only one *Notch2* gene copy per diploid cell [45]. However, the copy variations of Hs683 cells are distinct from both oligodendroglioma-derived and GBM-derived cells.

IDH mutations are prominent in oligodendrogliomas and appear early in gliomagenesis (i.e., prior to 1p/19q LOH) [31]; thus, 100% of tumors with complete 1p/19q co-deletion are mutated in IDH1 or IDH2 [46]. Therefore, DNA sequencing was undertaken to determine whether the OG cells harbored known IDH mutations. PCR of genomic DNA and sequencing of exon 4 containing codon R132 of IDH1 and codon R172 of IDH2 demonstrated that Hs683, OG33, and OG35 cells express wild-type IDH1 and IDH2 (Figure S2). HOG cells were previously reported to express wild-type IDH1/2 [47]. The wild-type IDH status in OG cells is not surprising given that only a 33% mutation rate is present in tumors bearing variable 1p/19q deletions [46] and the OG cells display the minimal 1p36/19q13 deletion. 

Finally, immunocytochemical profiling was performed (Figure 1C). In SCM, both OG33 and OG35 cells expressed NG2 and PDGFRα, two well-accepted markers of OPCs [44], but did not express Sox2 (Figure 1C) or Olig2 (not shown). In contrast, the cells abundantly express the mesenchymal marker CD44 and the oligodendrocyte-associated enzyme glutathione S-transferase π (GST π) [48]. In glioma, GST π is linked with drug resistance [49]. In DM, the OG cells expressed low levels of CNPase, but failed to express myelin basic protein (MBP) (Figure 1C) even when cultured in DM for up to 3 weeks (not shown). In contrast, Oli-Neu cells displayed MBP immunoreactivity after 3 days in SATO DM. Collectively, these data support the oligodendroglial origin of OG33 and OG35 cells.

### GTA induces growth arrest of oligodendroglioma cells, but not neural stem cells

**Figure 2:**
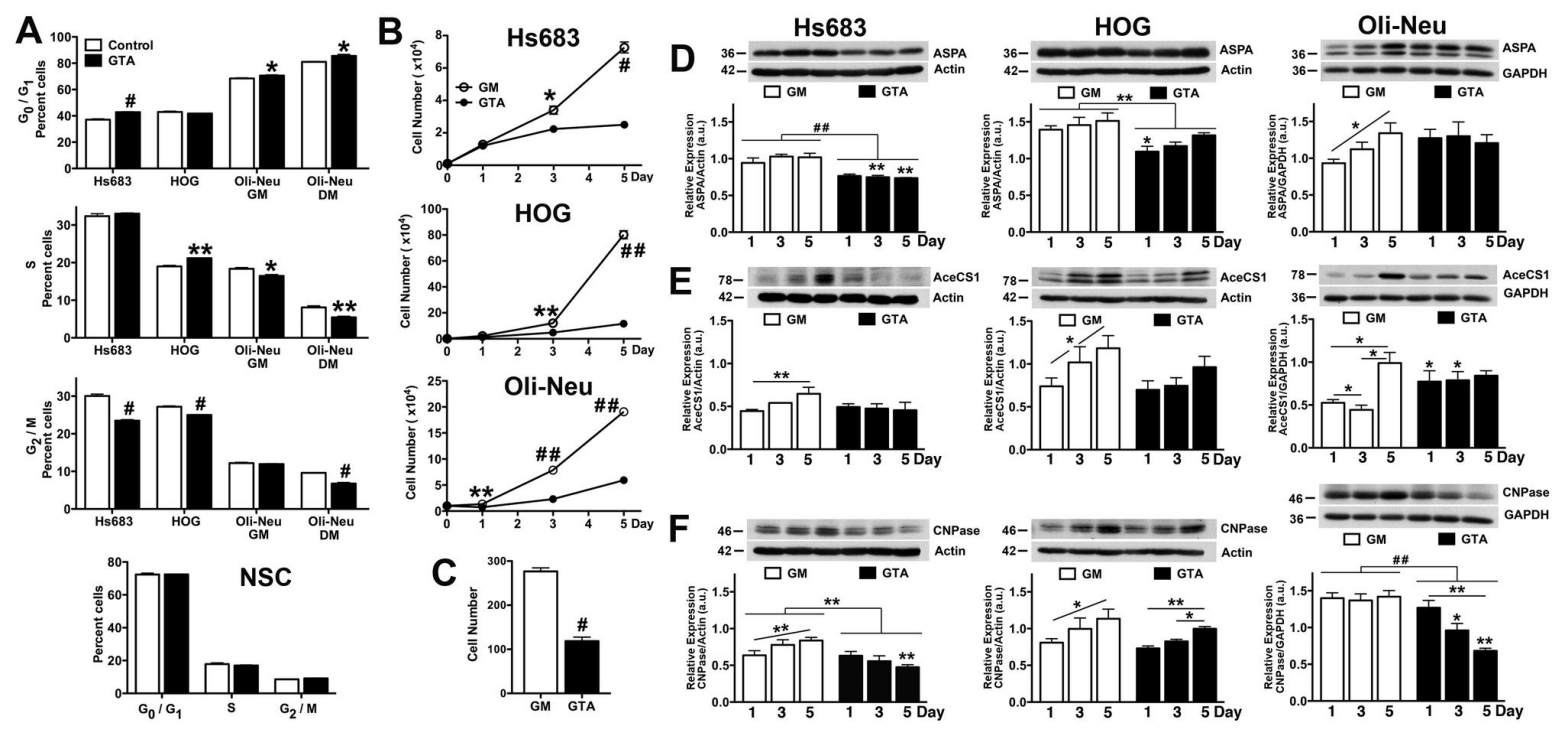
GTA reduces ASPA expression and inhibits oligodendrocyte differentiation. A) Cell cycle profiles of PI-labeled of established oligodendroglioma cells (Hs683, HOG), immortalized murine OPCs (Oli-Neu in growth medium [GM] and differentiation medium [DM]), and mouse neural stem cells (NSCs) after 24 hours of 0.25% GTA treatment. GTA induced G_0_ growth arrest of Hs683 and Oli-Neu cells, but not NSCs. B) Continuous growth in GTA (medium replenished every 48 hours) reduced cell growth dynamics, as assessed by unbiased trypan blue exclusion based cytometry. C) The mean number of adherent Oli-Neu cells 2 hours after plating in the absence or presence of GTA indicates that reduced adhesion contributes to the reduced growth of Oli-Neu cells. D-F) Western blots (25 μg whole cell lysate) and densitometric analysis of ASPA (D), AceCS1 (E) and CNPase (F) protein levels normalized to actin. D) GTA decreased ASPA levels in Hs683 and HOG cells, but not Oli-Neu cells. E) AceCS1 protein levels increased with time in culture in all lines and GTA blunted this temporal increase in Hs683 and HOG cells, but transiently increased AceCS1 levels in Oli-Neu cells. F) GTA decreased, or blunted the temporal increase of, CNPase protein levels in all lines examined, suggesting GTA does not reduce growth via the promotion of differentiation. *p < 0.05, **p ≤ 0.01, #p ≤ 0.001, ##p < 0.0001 unless otherwise indicated symbols represent significance relative to untreated cells at the same time point. n = 3 independent cultures.

Inasmuch as ASPA-mediated NAA catabolism to liberate acetate is reduced in glioma [15], the effect of GTA-mediated acetate supplementation on the growth and differentiation of established oligodendroglioma cells was tested (Figure 2). Twenty-four hours of treatment with 0.25% GTA induced G_0_/G_1_ growth arrest of Hs683 cells, but not HOG cells (Figure 2A). While GTA induced a modest G_0_/G_1_ growth arrest of Oli-Neu cells in both growth medium (GM) and DM, it had no effect on neural stem cells (NSCs) (Figure 2A). GTA treatment for 5 days significantly decreased cell growth in all lines, with the most rapidly dividing HOG cells most affected (Figure 2B). Even though Oli-Neu cells were plated onto PLL-coated dishes, decreased adhesion (Figure 2C) of these small cells may have contributed to their apparent growth reduction.

The effect of GTA-mediated acetate supplementation on ASPA protein levels, acetyl-CoA genesis (AceCS1 and AceCS2 protein levels), and oligodendrocyte differentiation (CNPase protein levels) was next assessed. The more rapidly dividing HOG cells possessed more ASPA (Figure 2D) and AceCS1 (Figure 2E) protein than Hs683 or Oli-Neu cells. GTA treatment decreased ASPA protein levels in Hs683 and HOG cells, but had no effect on Oli-Neu cells (Figure 2D). AceCS1 should be up-regulated to deal with the increased GTA-derived acetate load; however, GTA blunted the temporal increase of AceCS1 in Hs683 and HOG cells and transiently increased AceCS1 protein levels in Oli-Neu cells (Figure 2E). No significant regulation of AceCS2 protein levels was observed in the absence or presence of GTA (not shown). Since ASPA expression is positively correlated with oligodendrocyte differentiation *in vivo* [50], it was not surprising that the decreased ASPA levels induced by GTA in Hs683 and HOG cells was associated with decreased CNPase protein levels (Figure 2F). Although GTA did not alter ASPA levels in Oli-Neu cells, GTA significantly reduced CNPase protein levels (Figure 2F). 

Since ASPA undergoes cytoplasmic-nuclear shuttling [51] and its co-localization with AceCS1 may play a role in histone acetylation [50], the spatial distribution of these enzymes was examined (Figure S3). In control cultures, ASPA immunoreactivity was present in both the cytosol and nucleus, but appeared more prominent within the nucleus. Despite its demonstrated role in cytoplasmic lipid synthesis in peripheral tissues, AceCS1 immunoreactivity was predominantly nuclear in all cells and at all time points, similar to the expression pattern reported for the developing and adult rat brain [52]. Interestingly, GTA induced cytosolic accumulation of ASPA and a profound morphological alteration in Hs683 cells. GTA appeared to decrease the intensity of CNPase immunoreactivity (Hs683 and HOG cells) and the number of immunoreactive cells (Oli-Neu). Taken together, these data suggest that the promotion of differentiation does not underlie the growth inhibitory effects of GTA on established oligodendroglioma cell lines. 

### GTA promotes growth arrest greater than sodium acetate at comparable acetate concentrations

**Figure 3:**
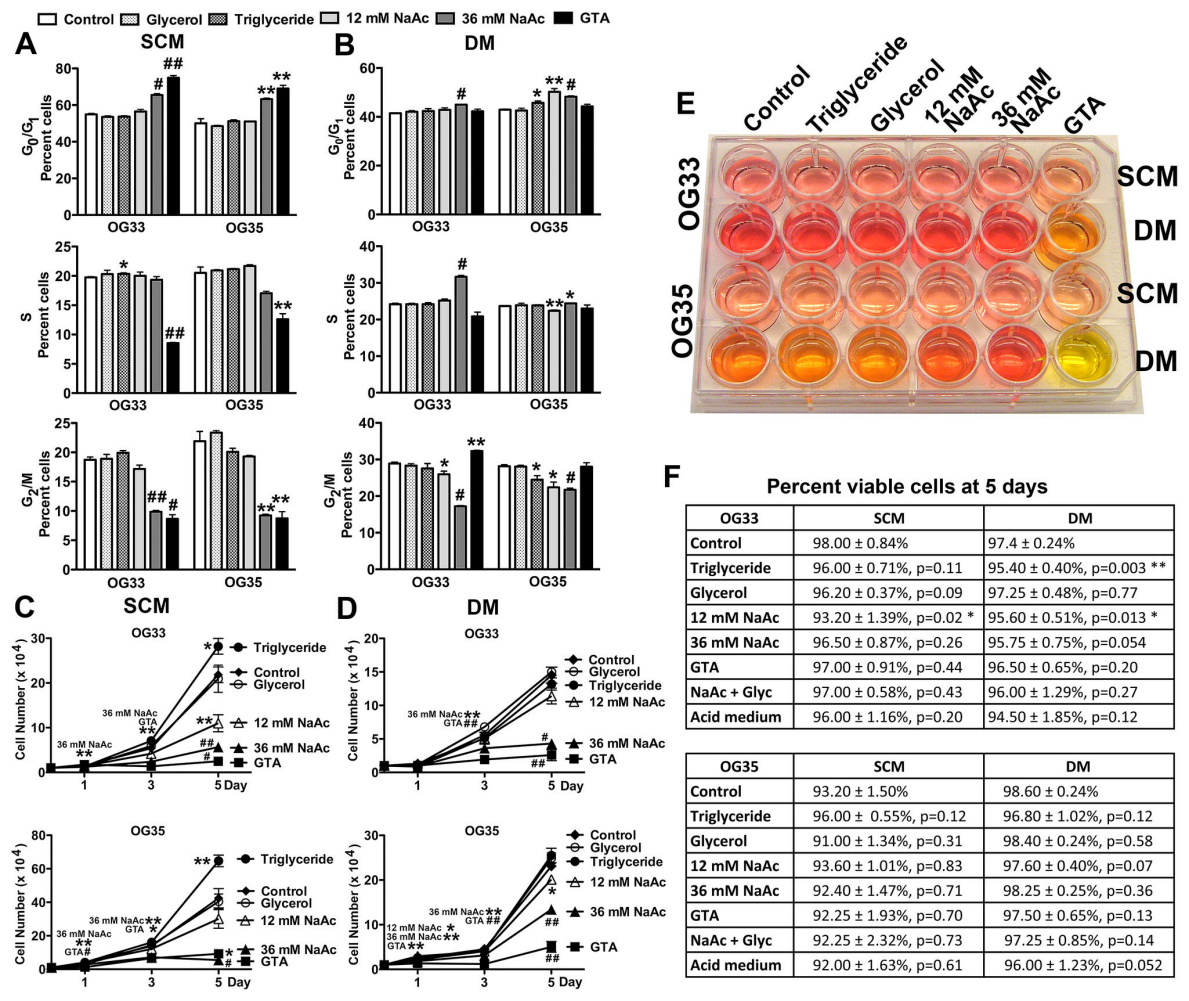
GTA inhibits OG33 and OG35 cell growth. A, B) Cell cycle profile of PI-labeled cells as floating spheres in stem cell medium (SCM) or in differentiation medium (DM) after 24 hours of treatment with 0.25% glycerol, 0.25% triglycerides (canola oil), 12 mM sodium acetate (NaAc), 36 mM sodium acetate (equivalent acetate to 0.25% GTA), or 0.25% GTA. In SCM (A), only 0.25% GTA reduced the proportion of dividing OG cells via G_0_ growth arrest. In DM (B), GTA had no effect on OG35 cells, but increased the percent of OG33 cells in S phase, without alterations in G_0_/G_1_ or G_2_/M. In contrast, 36 mM sodium acetate induced G_0_ growth arrest in both OG cells. C, D) Cells were treated for up to 5 days as described above with medium replenished every 48 hours. Growth dynamics were assessed by unbiased trypan blue exclusion based cytometry after 1, 3, and 5 days of treatment. In SCM (C), triglycerides promoted cell growth, glycerol had no effect on growth, and sodium acetate displayed a dose-dependent reduction in growth. GTA was more effective than 36 mM sodium acetate at growth reduction of OG33 cells. In DM (D), neither triglycerides nor glycerol affected growth, while sodium acetate displayed a dose-dependent growth reduction. GTA-mediated growth reduction was greater than sodium acetate in both OG cells. E) Photograph depicting representative medium coloration after 5 days in culture. F) The percent viable cells after 5 days of treatment was determined by unbiased trypan blue exclusion based cytometry. Only 12 mM sodium acetate and triglycerides in DM significantly reduced cell viability. *p < 0.05, **p ≤ 0.01, #p ≤ 0.001, ##p < 0.0001. n = 3 - 6.

Because GSCs exhibit altered metabolism relative to their non-stem tumor cell counterparts [53], the growth effect of GTA-derived acetate supplementation was investigated in primary oligodendroglioma-derived cells that exhibit self-renewal and tumorigenicity (grade II OG33 and grade III OG35). Since the active molecule of GTA is thought to be acetate [19,54-61], the growth inhibitory effects of GTA were compared to 36 mM sodium acetate (equivalent acetate to 0.25% GTA) and 12 mM sodium acetate (since 3 moles of acetate are derived per mole of GTA) as a positive control. GTA is cleaved by non-specific lipases and esterases in all cells into acetate and glycerol; thus, the growth effects of 0.25% glycerol alone and with 36 mM sodium acetate also were tested. As an intact molecule, GTA is a short chain triglyceride. There are no naturally occurring short chain triglycerides shorter than palmitate (C16), because it is the first fatty acid produced by fatty acid synthase, and is the precursor for all other longer chain fatty acids. Hence, all shorter triglycerides are synthetic, with GTA being the shortest. Rather than testing another synthetic triglyceride, we tested the growth effects of canola oil, a naturally occurring triglyceride that is frequently consumed because of its high levels of cholesterol-lowering fats (i.e., low in saturated fats and contains omega-6 and omega-3 fatty acids). In SCM, 24 hours of treatment with 0.25% GTA or 36 mM sodium acetate induced G_0_/G_1_ growth arrest, but only GTA decreased the percent of proliferating cells (Figure 3A). Glycerol, 12 mM sodium acetate, and triglycerides had no effect on cell growth. In DM, 36 mM sodium acetate, but not GTA, induced G_0_/G_1_ growth arrest of OG33 cells, while both 12 mM and 36 mM sodium acetate, but not GTA, induced G_0_/G_1_ growth arrest of OG35 cells, primarily due to fewer cells in G_2_/M (Figure 3B). These data indicate that short-term treatment with GTA induces growth arrest primarily in SCM and is more effective at decreasing proliferation than the equivalent moles of acetate.

Differences between GTA and sodium acetate became more apparent with continuous treatment for 5 days (Figures 3C-F). Both in SCM (Figure 3C) and DM (Figure 3D), sodium acetate induced a dose-dependent decrease in the growth of both OG cells. However, the growth decrement was greater for GTA treated cells than for sodium acetate treated cells. Treatment with 0.25% glycerol and 36 mM sodium acetate, equivalent to that generated by complete catalysis of 0.25% GTA, was not different than 36 mM sodium acetate alone (Figure S4), likely due to the fact that glycerol alone had no effect on cell growth. Strikingly, triglycerides increased the growth of OG cells in SCM (Figure 3C), but not DM (Figure 3D). Because GTA, but not 36 mM sodium acetate, resulted in significant medium acidification in DM between days 3 and 5 of treatment (Figure 3E; control OG33 cell medium pH = 7.30 ± 0.09, GTA treated cell medium: day 1 = 7.20 ± 0.11 p = 0.56, day 3 = 7.03 ± 0.15 p = 0.19, day 5 = 6.83 ± 0.09 p = 0.01; control OG35 cell medium pH = 7.28 ± 0.06, GTA treated cell medium pH: day 1 = 7.13 ± 0.09 p = 0.23, day 3 = 6.87 ± 0.18 p = 0.09, day 5 = 6.50 ± 0.10 p = 0.003), the growth effects of acidified medium was assessed (Figure S4). Incubation of cells with pH 6.5 medium from days 3-5 did not significantly after cell growth. The reduced growth of acetate treated cells was not due to reduced cell viability (Figure 3F). GTA also did not promote apoptosis, since no increased cleaved Poly ADP ribose polymerase immunoreactivity (Figure S5) or activated caspase-3 protein levels (not shown) were observed. Inasmuch as GTA-mediated growth arrest is greater in self-renewing cells in SCM and is more effective than sodium acetate, it suggests that GTA may exert functions distinct from its role as an acetate delivery vehicle. 

### ASPA and AceCS1 nuclear co-localization within OG cells is regulated during the cell cycle

**Figure 4:**
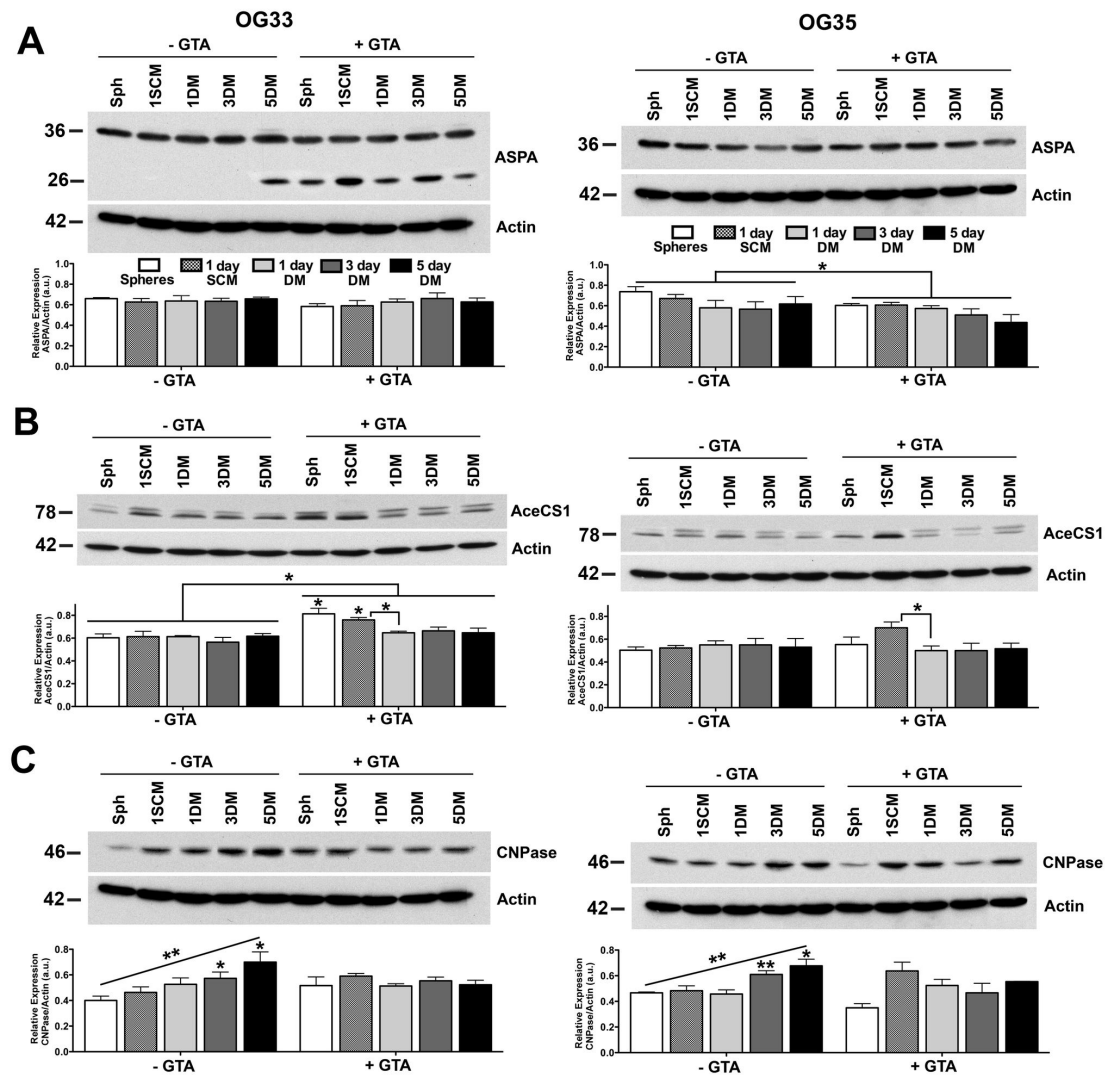
GTA inhibits OG33 and OG35 cell differentiation. Western blots (25 μg whole cell lysate) and densitometric analysis of ASPA (E), AceCS1 (F) and CNPase (G) protein levels normalized to actin. A) GTA treatment induced the presence of a novel immunoreactive Aspa species in OG33 cells (densitometry only shows the putative 36 kDa ASPA protein) and reduced ASPA protein levels in OG35 cells. B) GTA increased AceCS1 protein levels in OG33 cells only in SCM. C) GTA blunted the temporal increase in CNPase protein levels. *p < 0.05, **p ≤ 0.01, #p ≤ 0.001, ##p < 0.0001 unless otherwise indicated symbols represent significance relative to untreated cells at the same time point. n = 3 independent cultures.

To begin to address the mechanism of GTA-mediated growth arrest, we examined ASPA and AceCS1 protein levels and whether decreased growth was due to the promotion of differentiation. In OG33 cells, GTA had no effect on the abundance of the putative 36 kDa ASPA protein, but induced the expression of a novel 26 kDa immunoreactive species (Figure 4A). NAA and NAAG also enhanced the presence of this ASPA variant [18], suggesting its regulation by increased acetate bioavailability. The presence of the 26 kDa species in untreated cultures at 5 days may be due to metabolic exhaustion in confluent cultures. PCR of genomic DNA and sequencing of ASPA exon 5 was performed to determine whether this novel species could arise from one of the two most common ASPA mutations in individuals of Ashkenazi Jewish heritage (i.e., Y231X) [62], which results in a premature termination and a 26 kDa inactive protein (Figure S6). Codon 231 possessed a silent SNP that would not affect protein coding. Thus, the 26 kDa protein is not the product of premature termination within exon 5. The novel ASPA isoform in GTA treated OG33 cells was associated with increased AceCS1 protein levels (Figure 4B). In OG35 cells, GTA decreased ASPA protein levels (Figure 4A), but did not alter AceCS1 protein levels (Figure 4B). Similar to Hs683, HOG, and Oli-Neu cells, GTA blunted the temporal increase of CNPase in both OG33 and OG35 cells (Figure 4C), suggesting GTA-mediated growth suppression was not due to the promotion of differentiation. 

**Figure 5:**
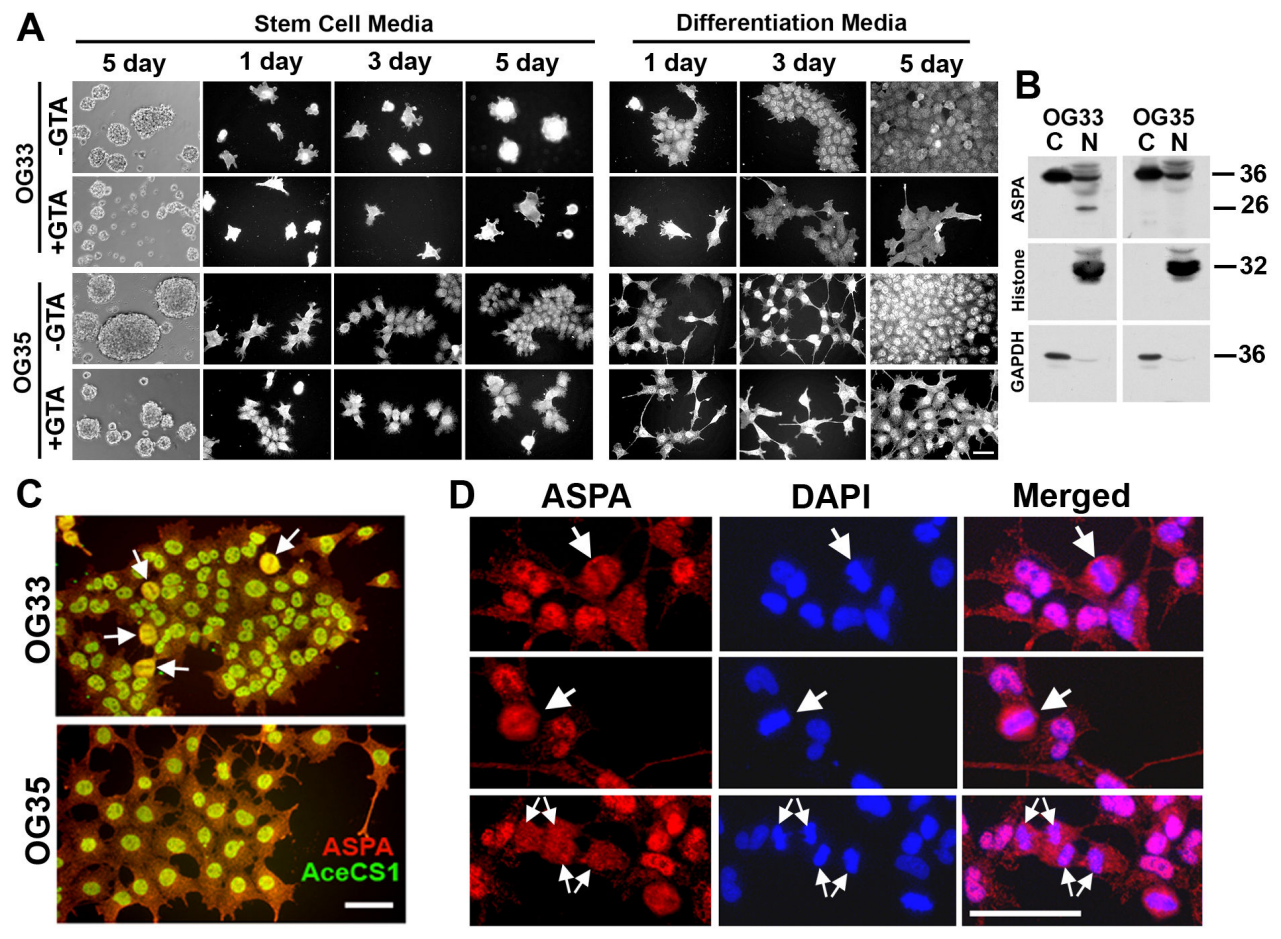
ASPA and AceCS1 nuclear co-localization is regulated during cell cycle progression. A) ASPA immunoreactivity was examined in the absence or presence of 0.25% GTA for 1, 3, or 5, days (2, 4, 6 days *in* *vitro*). In stem cell medium (on PLL-coated dish), OG33 cells grew as small adherent clusters while OG35 cells displayed a more splayed morphology. In both cases, ASPA was comparably expressed in the cytosol and nucleus. In differentiation medium, ASPA immunoreactivity was particularly enriched within the nucleus, and GTA reduced ASPA expression. B) Subcellular fractionation revealed that ASPA was more abundant in the cytosol (C), than nucleus (N), of both OG cell cultured in DM with 10 μM NAAG for 4 days. The lower molecular weight Aspa species induced by increased acetate bioavailability (by GTA, Figure 4A, and NAAG, shown here) exclusively partitioned to the nucleus. C) ASPA and AceCS1 were co-localized in the nucleus of untreated OG33 and OG35 cells cultured in DM for 3 days (4 days *in* *vitro*). Co-localization is most pronounced during metaphase (arrows). D) ASPA spatial localization in the nucleus of OG33 cells cultured in DM for 3 days (4 days *in* *vitro*) was present surrounding the metaphase equatorial plate and aligned chromosomes (DAPI staining, large single arrows). As sister chromatids separated in anaphase, ASPA immunoreactivity became more diffuse (small double arrows). Scale bar = 100 μm (A), 50 μm (C, D).

**Figure 6:**
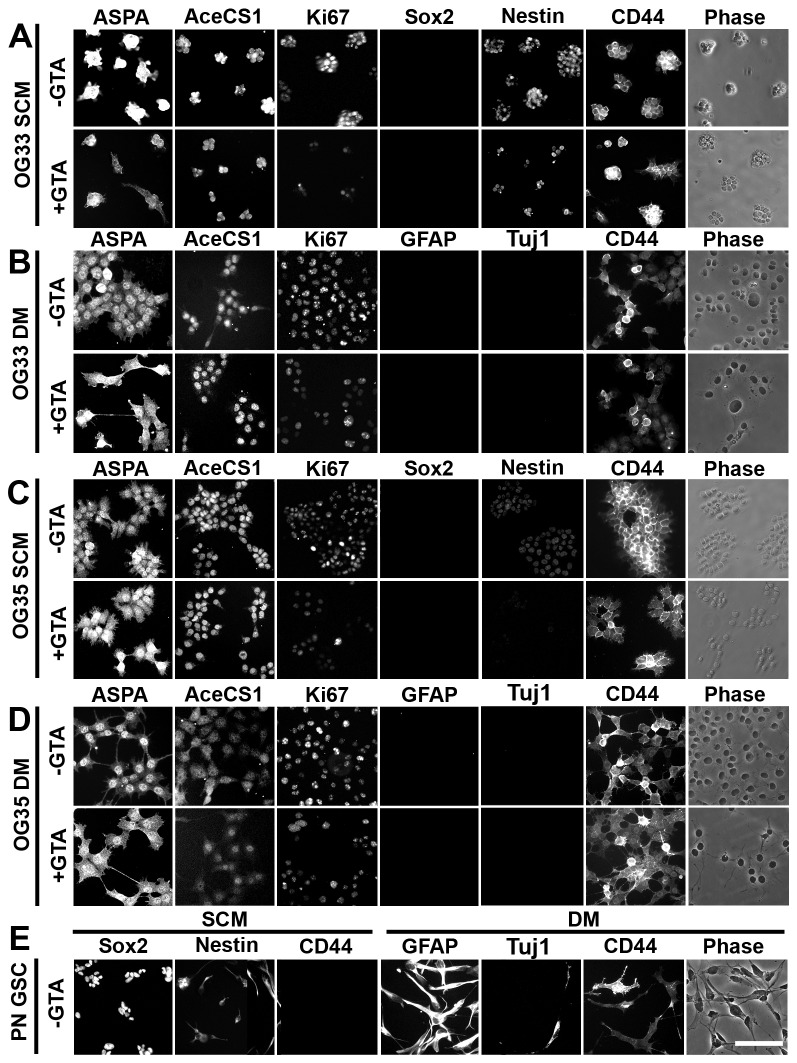
OG33 and OG35 cells express markers characteristic of a mesenchymal glioma subtype. OG33 (A, B) and OG35 (C, D) cells were cultured in stem cell medium (SCM) adherently on PLL-coated wells or differentiation medium (DM) in the absence or presence of 0.25% GTA for 3 days (4 days *in* *vitro*). Proneural (PN) GBM GSCs (E) expressed abundant SOX2 and nestin, but no detectable CD44 in SCM and differentiated into GFAP-positive astrocytes, Tuj1-positive neurons, and CNPase-positive oligodendrocytes (not shown) in DM. In contrast, OG33 and OG35 cells express low levels of nestin, no detectable SOX2, and abundant CD44 in SCM and do not generate GFAP-positive astrocytes or Tuj1 positive neurons in DM, features indicative of a mesenchymal glioma profile. Furthermore, in DM, OG33 and OG35 cells remain proliferative (i.e., Ki67-positive), while the proneural GSCs showed no Ki67-positive cells (not shown). ASPA appeared to be equally expressed in the cytosol and nucleus of both cells when grown in SCM, but, when grown in DM, ASPA appeared more abundantly expressed within the nucleus. In both growth conditions, AceCS1 was primarily expressed in the nucleus. GTA decreased overall ASPA and AceCS1 expression, but particularly within the nucleus (see also Figure 4A). Scale bar = 100 μm.

Since ASPA undergoes cytosolic-nuclear shuttling, it is critical to examine its spatial localization and not simply total protein levels. In SCM, ASPA appeared to be equally expressed within the nucleus and cytosol, while in DM, nuclear immunoreactivity appeared more intense (Figure 5A). However, subcellular fractionation did not support this cytological observation since full-length ASPA was more abundant in the cytosolic compartment; alternatively, ASPA was less easily extractable from the nuclear compartment (Figure 5B). Interestingly, the novel 26 kDa Aspa species in OG33 cells partitioned exclusively to the nucleus (Figure 5B). Strikingly, ASPA and AceCS1 co-localized within the nucleus of both OG cells (Figures 5C, 6) and were regulated during the cell cycle, with most prominent co-localization during metaphase (Figure 5D). GTA inhibited proliferation as revealed by decreased phospho-Histone H3 (Figure S5) and Ki67 (Figure 6) immunoreactivity. Furthermore, GTA appeared to decrease nuclear ASPA expression (Figures 5A, 6), suggesting that nuclear ASPA may promote cell proliferation. 

### Nuclear ASPA may contribute to the maintenance of an undifferentiated phenotype

**Figure 7:**
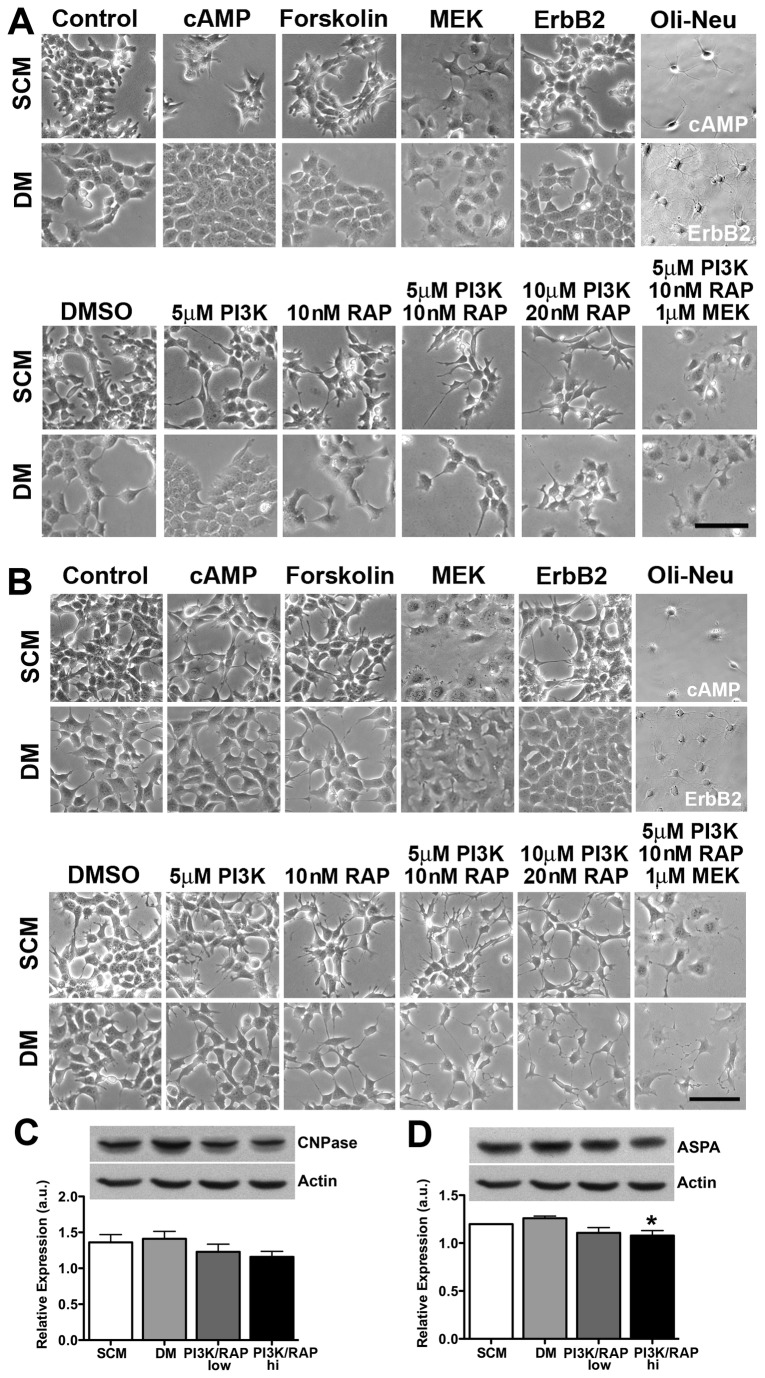
OG33 and OG35 cells are recalcitrant to differentiation. OG33 (A) and OG35 (B-D) cells were induced to differentiate over 6 days *in* *vitro* via activation of adenylyl cyclase (1 mM dibutyryl cAMP or 10 μM forskolin) or inhibition of MEK1/2 (1 μM PD035901), ErbB2 (1 μM PD174285), PI3K (5 μM and 10 μM LY294002), or mTOR (10 nM and 20 nM rapamycin [RAP]) signaling. DMSO (0.2% DMSO) was used as a control for PD174285, PD035901 and LY294002 treatments. Despite inhibition of PI3K-Akt-mTOR and ERK signaling pathways, both cells failed to differentiate into cells with oligodendroglial morphology (A, B) or increase immunoreactivity for GPAF or CNPase (not shown). MEK and ErbB2 inhibition actually increased OG35 proliferation in DM. While inhibition of PI3K-Akt-mTOR signaling induced morphological alterations in OG35 cells (B), this was not associated with increased CNPase (C) or ASPA (D) protein levels. In fact, ASPA expression decreased in the presence of 10 μM LY294002 and 20 nM rapamycin. Differentiation of Oli-Neu cells with cAMP for 4 days or inhibition of ErbB2 signaling for 2 days served as controls for oligodendrocyte cell morphology. DM - differentiation medium, SCM - stem cell medium. n = 3 independent cultures. *p < 0.05. Scale bar = 100 μm.

 Since the OG cells did not exhibit morphological or phenotypic alterations indicative of mature oligodendrocytes (Figure S7), their full differentiation capacity was assessed using agonists and antagonists of known signaling pathways dysregulated in glioma (Figure 7). Activation of adenylyl cyclase or inhibition of phosphoinositide 3-kinase, mammalian target of rapamycin, mitogen-activated protein kinase (Ras-Raf-MEK-ERK pathway), or ErbB2 signaling either alone or in combinations failed to promote differentiation of OG33 (Figure 7A) or OG35 (Figure 7B) cells. Although some treatments were associated with morphological alterations, these had little or no effect on the expression of CNPase (Figure 7C) or ASPA protein levels (Figure 7D), or any other marker examined (MBP, GFAP, Tuj1; not shown). 

Finally, based on their mesenchymal phenotype and the fact that GBM GSCs can differentiate into mesenchymal lineage cells [27,28,63], the adipogenic and osteogenic potential of the OG cells was investigated. The cells only survived a maximum of 5 days in adipogenic medium (not shown). In osteogenic medium, proliferation was slowed and cells underwent morphological alterations, but even after 3 weeks cells did not display osteoblastic differentiation (Figure S8). These results suggest that OG33 and OG35 cells represent differentiation arrested NG2/PDGFRα-positive tumor-initiating cells that share some properties of the mesenchymal subtype of glioma. 

### GTA promotes protein acetylation

**Figure 8:**
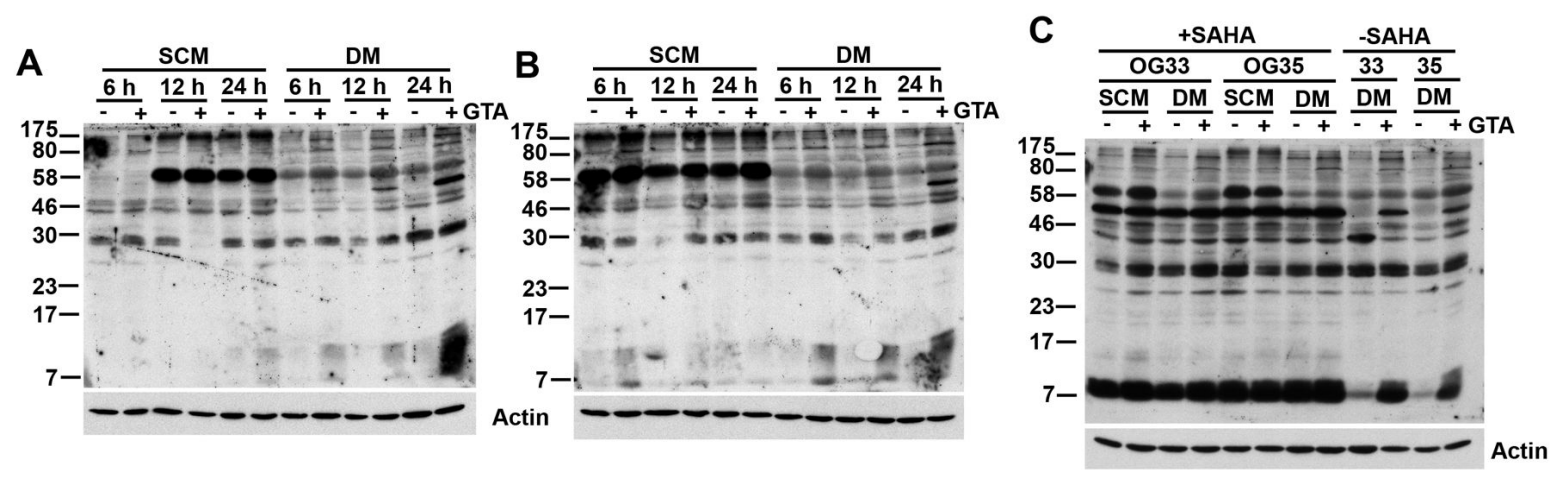
GTA increases protein acetylation of OG cells *in* *vitro*. OG33 (A) and OG35 (B) cells were treated with 0.25% GTA in the absence of an HDACi, the cells were harvested at the indicated time points, and analyzed by western blot analysis (25 μg protein, whole cell Triton Lysis Buffer lysate) with an antibody specific to acetylated lysine residues. Both cells displayed a time-dependent increase in acetylation of several proteins. C) Cells were treated with 0.25% GTA in the absence or presence of the HDACi SAHA (1 μM) for 24 hours and analyzed by western blot analysis (25 μg protein, whole cell RIPA Buffer lysate). There was a significant increase in the number of acetylated proteins in SAHA treated cells. Even in the absence of SAHA, GTA enhanced acetylation of several proteins, including those within the histone molecular weight range (H1-21.3kDa, H2a-14.3kDa, H2b-13.8kDa, H3-15.0kDa, and H4-11.3kDa).

Previously, we demonstrated that GTA enhanced temozolomide chemotherapeutic efficacy in orthotopic grafts of OG35 cells only when administered prior to, but not concurrent with or subsequent to, chemotherapy [15] and hypothesized that GTA promoted histone acetylation to promote an open, euchromatic state to allow increased chemotherapy access to DNA. Previous studies have demonstrated a role for GTA in histone acetylation *in vivo* [58,59]. To address which other proteins may be acetylated by GTA *in vitro*, OG cells were treated with 0.25% GTA, in the absence of an HDACi, for 6, 12 or 24 hours and the extent of lysine acetylation assessed by western blot analysis (Figures 8A, B). Even in the absence of an HDACi to prevent *in vitro* deacetylation, GTA increased acetylation of several proteins. The acetylation was specific since no increase in actin acetylation was detected. Inasmuch as acetylation is a dynamic process, it is possible that proteins acetylated by GTA could have become deacetylated either in culture or subsequent to extraction in triton lysis buffer. Therefore, cells were treated with 0.25% GTA in the absence or presence of the HDACi SAHA (1 μM) for 24 hours, extracted with RIPA buffer to increase histone protein extraction, and analyzed by western blot analysis. Not unexpectedly, there was a significant increase in the number of acetylated proteins in SAHA treated cells. Even in the absence of SAHA, GTA increased protein acetylation, including several within the molecular weight range for histones H3 and H4 (i.e., H3-15.0kDa, and H4-11.3kDa). Preliminary mass spectrometry analysis indicates increased acetylation of histone H4K8, H4K12, and H4K16 as well as other proteins involved in cell cycle regulation (Lam & Jaworski, unpublished observation). Moreover, recent microarray data revealed that GTA significantly decreased expression of several histone isoforms in OG33 cells, which expresses a novel nuclear restricted ASPA isoform, but not OG35 cells expressing only full length ASPA, even though GTA induced comparable cytostatic growth arrest in both cells (Long & Jaworski, unpublished observation). Collectively, our data suggest that GTA exerts growth inhibitory effects via an epigenetic mechanism without promoting differentiation and that cells with the novel ASPA isoform may utilize the acetate differently than those only expressing full-length ASPA. 

## Discussion

Due to the decreased incidence and better prognosis of oligodendroglioma relative to GBM, few studies have endeavored to generate oligodendroglioma cell lines. Although OG33 and OG35 cells were derived from primary oligodendroglioma tumors (grade II and III, respectively) using a well-established protocol for the generation of GSCs [24], these cells exhibit only some GSC and oligodendroglioma features.

Although the OG cells exhibit 1p/19q LOH, *O* ^*6*^ *-methylguanine-methyltransferase* promoter hypermethylation, self-renewal, and form aggressive orthotopic tumors [15], they express wild-type IHD1/2. Since all four oligodendroglioma lines used in the study expressed wild-type IDH1/2, it is possible that cells harboring IDH mutations cannot be propagated *in vitro* [64] because cell culture selects for the more aggressive wild-type IDH1/2 cells. In fact, cells must be transfected to express mutant IDH1/2 to study their function [47]. Interestingly, this study showed that NAAG was reduced 50-fold and 8.3-fold in HOG cells expressing IDH1 and IDH2 mutants, respectively. Therefore, it would be intriguing to assess whether ASPA protein levels, ASPA subcellular localization, or GTA-mediated growth arrest are altered in cells bearing IDH1/2 mutations.

Another distinctive feature of the OG cells is their lack of differentiation into astrocytes or mature MBP-positive oligodendrocytes, as would be expected of OPCs, a proposed glioma cell of origin [36-39]. Oligodendrogliomas exhibit a proneural genetic signature [40,41] and the OG cells expressed NG2 and PDGFRα; however, they did not express the proneural markers Sox2, CD133, or Olig2 and only expressed low levels of Notch1 and nestin. CNS microvasculature-associated pericytes also express NG2 and PDGFRα and form multicellular spheres in growth factor containing medium, like OG cells. However, unlike the OG cells, pericytes exhibit multipotential differentiation capacity in serum containing medium [65]. An alternative explanation is that OG cells simply reflect differentiation-arrested NG2-positive OPCs which are the predominant cell type in low-grade gliomas [38] and display significant tumor-initiating potential [37] similar to the OG cells [15]. However, the OG cells abundantly express the mesenchymal markers CD44, BCL2A1, Wilms Tumor 1, similar to the recently described transitional glioma phenotype [66].

Considerable effort was expended to differentiate OG cells into mature MBP-positive oligodendrocytes. In addition to the pharmacological manipulations reported here, we also attempted to differentiate the cells in a proprietary OPC differentiation medium (ScienCell) or SATO differentiation medium, DMEM/FBS or neurobasal A with B27 supplement in which each medium was supplemented with all-trans retinoic acid, TGFβ1, PDGFα in the absence or presence of bFGF, or 3,5-tri-iodothyronine in the absence or presence of ascorbic acid or CNTF (unpublished observation). Although many treatments induced morphological alterations, the cells failed to adopt the stereotypical mature oligodendrocyte morphology and did not express MBP, the most commonly used marker of mature oligodendrocytes. We do, however, acknowledge the limitation of using MBP as the sole mature oligodendrocyte marker. Nevertheless, the concept that glioma is typified by the presence of highly de-differentiated cells that exhibit the capacity for differentiation, but have profound impairment in terminal differentiation due to a bias toward self-renewal is not a new one. Indeed, many of the molecules that promote stemness have been identified, including, WNT, sonic hedgehog, Notch, Myc, p53, Pten, and A2BP1 [67-69]. It is possible that ASPA, particularly nuclear ASPA, may similarly promote an undifferentiated state.

While ASPA protein levels are significantly reduced in oligodendroglioma and astrocytoma tumors relative to normal brain [15], ASPA levels in oligodendroglioma-derived cells *in vitro* are comparable to Oli-Neu OPCs. Although Oli-Neu cells differentiate into mature oligodendrocytes with high fidelity, thus permitting the investigation of ASPA in OPC differentiation, these cells are oncogene addicted due to constitutive ErbB activation. Hence, we acknowledge the limitation of using Oli-Neu cells as "normal" non-transformed OPCs with which to compare oligodendroglioma cells. For example, in Oli-Neu cells, ASPA levels were increased with time in culture even though CNPase levels did not increase, while in human oligodendroglioma cells, ASPA levels did not change even though CNPase levels increased. ASPA plays a well-accepted role in oligodendrocyte differentiation *in vivo* [70-75], suggesting the need for neuron-glial interaction. However, co-culture of OG cells with SH-SY5Y neuroblastoma or PC12 pheochromocytoma cells also failed to promote OG differentiation (unpublished observation). Hence, the basis for the differences in ASPA regulation in Oli-Neu and OG cells *in vitro* with OPCs *in vivo* is not known at the present time.

Although best characterized as an oligodendroglial cytosolic enzyme that supports lipogenesis for myelination, evidence indicates ASPA exerts additional nuclear functions. However, its nuclear function is a topic of considerable speculation since ASPA undergoes cytosolic-nuclear shuttling via an unknown mechanism and nuclear ASPA displays reduced catalytic activity towards NAA relative to cytosolic ASPA [51]. The nuclear co-localization of ASPA and AceCS1 is well documented [50-52,76], but to our knowledge this is the first report to document that the subnuclear spatial localization of ASPA is regulated during the cell cycle. Despite high salt, sonication, and heat used to extract nuclear ASPA, a substantial amount remained in the insoluble pellet, suggesting association with DNA/chromatin. Unfortunately, at present this precludes quantitative analysis of ASPA subcellular partitioning following GTA treatment. Because normal OPCs exhibit more extensive histone acetylation than mature oligodendrocytes [77] and OG cells remain highly proliferative even after long-term growth in DM, we propose that nuclear ASPA may promote histone acetylation to maintain a proliferative progenitor-like state. This is supported by the abundant presence of ASPA and NAA in murine OPCs as early as embryonic day 12.5 [72], suggesting ASPA subserves functions distinct from NAA-mediated catalysis for myelination, as proposed earlier [56]. 

Previously, we examined the regulation of ASPA in OG cells using NAA and NAAG as an acetate source, but discovered these metabolites increased cell growth [18]; thus an alternative acetate source was sought. GTA is primarily viewed as a delivery vehicle for acetate [19,54-61]. However, similar to an *in vivo* study demonstrating that GTA was a more effective acetate supplement than calcium acetate [54], we showed that 0.25% GTA (~12 mM) displayed greater growth inhibition than 36 mM sodium acetate plus 0.25% glycerol, equivalent to that generated by complete catalysis of 0.25% GTA. Although decreased absorption was postulated as the basis for reduced brain acetate levels following *in vivo* calcium acetate treatment, this should not be an issue in cultured cells since sodium acetate is taken up by monocarboxylate transporters which are abundantly expressed and widely distributed since they transport important metabolic compounds including lactate, pyruvate, and ketone bodies [78]. The lack of growth inhibition by glycerol alone or increased growth inhibition when 36 mM sodium acetate was combined with 0.25% glycerol is likely not due to deficits in cellular uptake. Aquaporins 3, 7, 9, and 10, which are subcategorized as aquaglyceroporins, are permeable not only to water, but also to small solutes such as glycerol, urea, and monocarboxylates [79]. Only aquaporin 1, 4, and 9 are expressed in the brain [80]. Since aquaporin 9 is expressed in NSCs and their glial progeny [81], it is the most likely glycerol transporter in our cells. Whereas GTA and 36 mM sodium acetate reduced cell growth, only GTA promoted media acidification, again highlighting differences between GTA and sodium acetate. Extracellular acidification rate *in vitro* is primarily driven by lactic acid release resulting from accelerated glycolysis of tumor cells (i.e., the Warburg effect). Tumor cells become adapted for growth and survival in low pH conditions via increased drug resistance [82] and resistance to autophagy [83] and p53-mediated apoptosis [84]. Our results suggest that, in contrast to dichloroacetate that shifts metabolism from glycolysis to oxidative phosphorylation [85], GTA promotes glycolysis rather than acetate-mediated AceCS2-dependent acetyl-CoA production and oxidative phosphorylation. The increased acidification of the more rapidly dividing OG35 cells than OG33 cells appears to support this hypothesis. Although a recent study demonstrated that GTA does not alter mitochondrial biogenesis under physiological conditions *in vivo* [61], whether GTA initially promoted oxidative metabolism and then resulted in selection of the most glycolytically converted transformed cells needs to be established. 

GTA has been reported to increase histone acetylation at H3K9, H4K8, and H4K16 [59] via inhibition of HDAC activity and expression [58]. Our mass spectrometry analysis of GTA treated OG cells has identified several candidate cell cycle regulatory proteins that may contribute to GTA-mediated growth arrest. As a short-chain fatty acid, GTA may not only serve as an acetate source, but an HDACi, similar to sodium butyrate. Unfortunately, our approach does not permit determining whether the increased acetylation observed in GTA treated OG cells was the direct result of GTA-mediated acetylation or an indirect affect via the prevention of deacetylation. Studies utilizing ^13^C-labeled GTA and mass spectroscopy will need to be performed to discern the relative contribution of GTA to acetylation promotion and deacetylation inhibition. 

GTA is an FDA approved food additive with “generally regarded as safe” status that has been tested for parenteral nutrition in a wide variety of species with no adverse effects [86]. Furthermore, infants with Canavan Disease have been chronically treated with high dose GTA (4.5 g/kg/day, similar to that used in our orthotopic graft study [15]) and showed no hepatotoxicity or significant side effects [87]. Since GTA promotes cytostatic growth arrest of oligodendroglioma-derived cells, but not normal cells, and increases protein acetylation without overt toxicity, we assert that identification of GTA's targets and further investigations of GTA as a chemotherapeutic adjuvant are warranted. 

## Supporting Information

Methods S1 **Details PCR primer sequences used for rtPCR profiling as well as the source and concentration of antibodies used for immunocytochemistry and western blot analysis.**
(DOCX)

Table S1 **Cell line validation by STR profiling.** DNA fingerprinting confirmed the STR profile of the commercially available Hs683 cell line (www.ATCC.org). Not surprisingly, the STR profiles of the non-commercial cell lines derived from primary human tumor specimens failed to correspond to known fingerprints in the CLIMA database (http://bioinformatics.istge.it/clima/). Although OG33 cells were derived from a male, STR profiling failed to identify the Y chromosome version of amelogenin. Although HOG cells and OG33 cells exhibit identical STR profiles, Nsp copy number mapping (Figure S1) revealed these cells are not identical.
(DOCX)

Figure S1 **Nsp copy number karyotype maps.**
(PDF)

Figure S2 **Hs683, OG33, and OG35 cells express wild-type IDH1 and IDH2.** PCR amplification and sequencing of genomic DNA corresponding to exon 4 of IDH1 and IDH2 was performed to determine whether the cells harbored mutations in IDH1 (R132H: CAT, R132C: TGT, R132G: GGT, or R132S: AGT) or IDH2 (R172G: GGG, R172K: AAG, R172W: TGG or R172M: ATG). All cell lines possessed wild-type IDH1 (R132: CGT) and IDH2 (R172: AGG) sequences.
(TIF)

Figure S3 **GTA alters morphology of oligodendroglioma cells and OPCs.** Cells were plated in GM for 24 hours prior to treatment with 0.25% GTA for 1, 3, or 5 days (i.e., 2, 4, and 6 days *in* *vitro*). In addition to its stereotypical cytosolic localization, abundant ASPA immunoreactivity was present within the nucleus of all three cell lines. GTA induced cytosolic accumulation of ASPA and a profound morphological alteration in Hs683 cells, decreased ASPA immunoreactivity in HOG cells, and increased cytosolic ASPA accumulation in Oli-Neu processes. Similar to ASPA, AceCS1 was abundant in the nucleus and increased with time in culture in all three cell lines. CNPase immunoreactivity was modestly increased with time in culture in Hs683and HOG cells and GTA reduced labeling. Oli-Neu cells with a branched, differentiated morphology increased with time in culture even in SATO GM. GTA reduced the frequency of differentiated CNPase-positive Oli-Neu cells. Scale bar = 100 µm.
(TIF)

Figure S4 **GTA mediated growth arrest is greater than the combination of 36 mM sodium acetate and 0.25% glycerol.** OG33 and OG35 cells were plated in stem cell medium (SCM) as floating spheres or in differentiation medium (DM) in the absence or presence of 0.25% GTA, 36 mM sodium acetate (NaAc, equivalent acetate to 0.25% GTA), or 36 mM sodium acetate plus 0.25% glycerol (equivalent to what is derived from 0.25% GTA). Because GTA treatment in DM resulted in medium acidification of OG33 and, to a greater extent, OG35 cells between days 3 and 5, cells were treated on day 3 with acidified medium (pH 6.5, the maximum attained in OG35 GTA treated cells). Medium was replenished every 48 hours. Cell growth was determined after 1, 3, and 5 days of treatment by unbiased trypan blue based cytometry. Overall, 0.25% GTA more effectively reduced cell growth than 36 mM sodium acetate, with OG35 cells in SCM being the notable exception. Cell growth was comparable with 36 mM sodium acetate alone and the addition of 0.25% glycerol. The addition of acidified medium at day 3 did not alter cell growth.
(TIF)

Figure S5 **GTA does not reduce cell growth via apoptosis.** Cells were grown in the absence or presence of 0.25% GTA in DM for 5 days and proliferation (phospho-Histone H3 [Ser10]), apoptosis (cleaved Poly ADP ribose polymerase, Asp214]), and cytoskeletal architecture (α-tubulin) were examined with the PathScan multiple immunofluorescence kit. GTA induced cytostasis via reduced proliferation, but not increased apoptosis. Scale bar = 100 μm.
(TIF)

Figure S6 **OG33 cells express wild-type ASPA.** PCR amplification and sequencing of genomic DNA corresponding to exon 5 of ASPA was performed to determine whether the novel 26 kDa ASPA immunoreactive species present in OG33 cells (Figure 4) could arise from the most common ASPA mutation (i.e., Y231X), which results in a premature termination and a 26 kDa inactive protein. Codon 231 possessed a silent single nucleotide polymorphism (C/T) that would not affect protein coding. Thus, the 26 kDa protein in OG33 cells does not originate from premature termination within exon 5.
(TIF)

Figure S7 **OG cells express markers of immature and young oligodendrocytes, but not myelin basic protein (MBP), a marker or more mature oligodendrocytes.** OG33 and OG35 cells were plated in stem cell medium (SCM) adherently on PLL-coated wells or differentiation medium (DM) for 5 days. Both cells abundantly express NG2, PDGFRα, and CNPase in SCM and retain expression in DM; however, cells failed to express detectable MBP immunoreactivity. Scale bar = 100 μm.
(TIF)

Figure S8 **OG cells do not undergo osteoblastic differentiation.** OG33 (A) and OG35 (B) cells were plated in DM (DMEM with 10% FBS) or osteogenic medium for up to 3 weeks. Medium was replenished every 3-4 days. After 5 days in osteogenic medium, reduced cell growth and altered cell morphology was apparent relative to cells in DM. However, in 3 week cultures, no Alizarin Red S positive cells or mineralized foci were detected (not shown). Furthermore, no increased CNPase immunoreactivity was observed and cells did not display immunoreactivity for myelin basic protein (MBP) or glial fibrillary acidic protein (GFAP). Thus, even protracted growth under differentiation conditions failed to induce mature glial or mesenchymal differentiation. Scale bar = 100 μm.
(TIF)
