# Supplementary material for: Acetate Supplementation Induces Growth Arrest of NG2/PDGFRα-Positive Oligodendroglioma-Derived Tumor-Initiating Cells
Source: PLoS One. 2013 Nov 20;8(11):e80714. doi: 10.1371/journal.pone.0080714 (PMC3835562; doi:10.1371/journal.pone.0080714)
Supplement: Methods S1 — Details PCR primer sequences used for rtPCR profiling as well as the source and concentration of antibodies used for immunocytochemistry and western blot analysis. (DOCX) [file pone.0080714.s001.docx]

**­­Supplementary Methods**

**Reverse transcription PCR Primer Sequences**

| **Gene** | **Primer Sequence** | Melting temp | GC content | Product size |
| --- | --- | --- | --- | --- |
| IDH1 | Forward: 5'-CTCCTGATGAGAAGAGGGTTG-3' | 54.6 °C | 52.3% |  |
|  | Reverse: 5'-TGGAAATTTCTGGGCCAT-3' | 51.8 °C | 44.4% | 248 bp |
|  |  |  |  |  |
| IDH2 | Forward: 5'-TGGAACTATCCGGAACATCC-3' | 54.0 °C | 50.0% |  |
|  | Reverse: 5'-ACTCTGTGGCCTTGTACTGC-3' | 57.3 °C | 55.0% | 228 bp |
|  |  |  |  |  |
| ASPA | Forward: 5'-AGTGGAGATGGGGTTCACC-3' | 62.3 °C | 57.9% |  |
|  | Reverse: 5'-TACGCGAAGTGCTGTATGAGC-3' | 62.6 °C | 52.4% | 362 bp |
|  |  |  |  |  |
| CD133 | Forward: 5'-ACTCCCATAAAGCTGGACCC-3' | 62.4 °C | 55.0% |  |
|  | Reverse: 5'-TCAATTTTG GATTCATATGCCTT-3' | 55.6 °C | 30.4% | 133 bp |
|  |  |  |  |  |
| Notch1 | Forward: 5'-AGTGTGAAGCGGCCAATG-3' | 59.9 °C | 55.6% |  |
|  | Reverse: 5'-ATAG TCTGCCACGCCTCTG-3' | 62.3 °C | 57.9% | 149 bp |
|  |  |  |  |  |
| SOX2 | Forward: 5'-ACCGGCGGCAACCAGAAGAACAG-3' | 68.1 °C | 60.9% |  |
|  | Reverse: 5'-GCGCCGCGGCCG GTATTTAT-3' | 66.6 °C | 65.0% | 255 bp |
|  |  |  |  |  |
| PDGFRα | Forward: 5'-CTCCTGAGAGCATCTTTGAC 3' | 60.4 °C | 50.0% |  |
|  | Reverse: 5'-GTAGAATCCACCATCATGCC 3' | 60.4 °C | 50.0% | 124 bp |
|  |  |  |  |  |
| Nestin | Forward: 5'-CAGCGTTGGAACAGAGGTTG-3' | 62.4 °C | 55.0% |  |
|  | Reverse: 5'-GACATCTTGAGGTGCGCCAG-3' | 64.5 °C | 60.0% | 163 bp |
|  |  |  |  |  |
| Olig2 | Forward: 5'-CTCCTCAAATCGCATCCAGA-3' | 60.4 °C | 50.0% |  |
|  | Reverse: 5'-AGAAAAAGGTCATCGGGCTC-3' | 60.4 °C | 50.0% | 147 bp |
|  |  |  |  |  |
| BCL2A1 | Forward: 5'-ATGGATAAGGCAAAACGGAG-3' | 58.4 °C | 45.5% |  |
|  | Reverse: 5'-TGGAGTGTCCTTTCTGGTCA-3' | 60.4 °C | 50.0% | 150 bp |
|  |  |  |  |  |
| WT1 | Forward: 5'-TTAAAGGGAGTTGCTGCTGG-3' | 60.4 °C | 50.0% |  |
|  | Reverse: 5'-GACACCGTGCGTGTGTATTC-3' | 62.4 °C | 55.0% | 141 bp |
|  |  |  |  |  |
| CD44 | Forward: 5'-CCCAGATGGAGAAAGCTCTG-3' | 62.4 °C | 55.0% |  |
|  | Reverse: 5'-ACTTGGCTTTCTGTCCTCCA-3' | 60.4 °C | 50.0% | 138 bp |
|  |  |  |  |  |
| GAPDH | Forward: 5'-GAAGGTGAAGGTCGGAGTCA-3' | 62.4 °C | 55.0% |  |
|  | Reverse: 5'-TTGAGGTCAATGAAGGGGTC-3' | 60.4 °C | 50.0% | 117 bp |

**Antibodies**

The following antibodies were used: rabbit anti-human ASPA (GTX13389 GeneTex, Irvine, CA; 7,500X for western blot, 500X for immunocytochemistry), rabbit anti-mouse AceCS1 (15,000X western blot) {Ariyannur, 2010 #4181}, rabbit anti-human AceCS1 150X immunocytochemistry; #6516-1 Epitomics; Burlingame, CA), rabbit anti-acetylated lysine (1,000X; #9441 Cell Signaling Technology; Danvers, MA), mouse anti-CD44 (2,000X, #5640 Cell Signaling), mouse anti-porcine glial fibrillary acidic protein (GFAP, 2,500X blots; 5,000 immuno; G3893 Sigma), rabbit anti-*E. coli* Glutathione S-Transferase pi (GSTπ, 800X; AB8902 Millipore; Billerica, MA), rabbit anti-human Ki67 (50X, ab833 Abcam; Cambridge, MA), rat anti-bovine myelin basic protein (MBP, 25X; ab7349 Abcam), mouse anti-human nestin (1,000X, ab22035 Abcam), rabbit anti-rat NG2 (250X, AB5320 Millipore), and rabbit anti-human Sox2 (1,000X, ab97959 Abcam), and mouse anti-human neuron-specific βIII tubulin (Tuj1, 3,000X; ab7751 Abcam). The expression of 2',3'-Cyclic-nucleotide 3'-phosphodiesterase (CNPase) was assessed immunocytochemically with 3 distinct antibodies: rabbit anti-mouse (250X, sc-30158 Santa Cruz), mouse anti-human (250X, ab6319 Abcam), and rabbit anti-human (100X, #5664 Cell Signaling). The expression of platelet-derived growth factor receptor-α (PDGFR-α) was assessed with 2 distinct antibodies: rabbit anti-human (100X, sc-338 Santa Cruz) and rabbit polyclonal (800X, #5241 Cell Signaling). Goat anti-human actin (1,000X, sc-1616), mouse anti-human histone H1 (250X, sc-8030) and rabbit anti-human glyceraldehyde-3-phosphate dehydrogenase (GAPDH, 5,000X: sc-25778) were obtained from Santa Cruz Biotechnology. CNPase expression by western blot analysis was assessed using rabbit anti-mouse (5,000X, Santa Cruz). Phospho-Histone H3 (Ser10), cleaved Poly ADP ribose polymerase (PARP, Asp214), and α-tubulin were co-localized using the PathScan apoptosis and proliferation multiple immunofluorescence kit (#7851 Cell Signaling). Species-specific HRP- (3,000X), Cy3- (500X) and Cy2- (100X) conjugated secondary antibodies were obtained from Jackson ImmunoResearch (West Grove, PA).
