# Supplementary material for: Acetate Supplementation Induces Growth Arrest of NG2/PDGFRα-Positive Oligodendroglioma-Derived Tumor-Initiating Cells
Source: PLoS One. 2013 Nov 20;8(11):e80714. doi: 10.1371/journal.pone.0080714 (PMC3835562; doi:10.1371/journal.pone.0080714)
Supplement: Table S1 — Cell line validation by STR profiling. DNA fingerprinting confirmed the STR profile of the commercially available Hs683 cell line (www.ATCC.org). Not surprisingly, the STR profiles of the non-commercial cell lines derived from primary human tumor specimens failed to correspond to known fingerprints in the CLIMA database (http://bioinformatics.istge.it/clima/). Although OG33 cells were derived from a male, STR profiling failed to identify the Y chromosome version of amelogenin. Although HOG cells and OG33 cells exhibit identical STR profiles, Nsp copy number mapping (Figure S1) revealed these cells are not identical. (DOCX) [file pone.0080714.s002.docx]

|  | Hs683 | HOG | OG33 | OG35 |
| --- | --- | --- | --- | --- |
| STR Locus | Alleles | Alleles | Alleles | Alleles |
| TH01 | 6, 8 | 9 | 9 | 9 |
| D21S11 | 27, 33.2 | 30 | 30 | 29, 30 |
| D5S818 | 11, 12 | 12, 13 | 12, 13 | 12, 13 |
| D13S317 | 8, 12 | 12 | 12 | 12 |
| D7S820 | 11 | 10 | 10 | 10 |
| D16S539 | 9, 10 | 9 | 9 | 9 |
| CSF1PO | 9, 13 | 12 | 12 | 12 |
| TPOX | 8, 11 | 9 | 9 | 9 |
| vWA | 18, 20 | 15 | 15 | 15 |
| Amelogenin | X, Y | X | X* | X |

**Supplementary Table 1 Cell Line Validation by STR Profiling**
