# Supplementary material for: Acetate Supplementation Induces Growth Arrest of NG2/PDGFRα-Positive Oligodendroglioma-Derived Tumor-Initiating Cells
Source: PLoS One. 2013 Nov 20;8(11):e80714. doi: 10.1371/journal.pone.0080714 (PMC3835562; doi:10.1371/journal.pone.0080714)
Supplement: Figure S1 — Nsp copy number karyotype maps. (PDF) [file pone.0080714.s003.pdf]

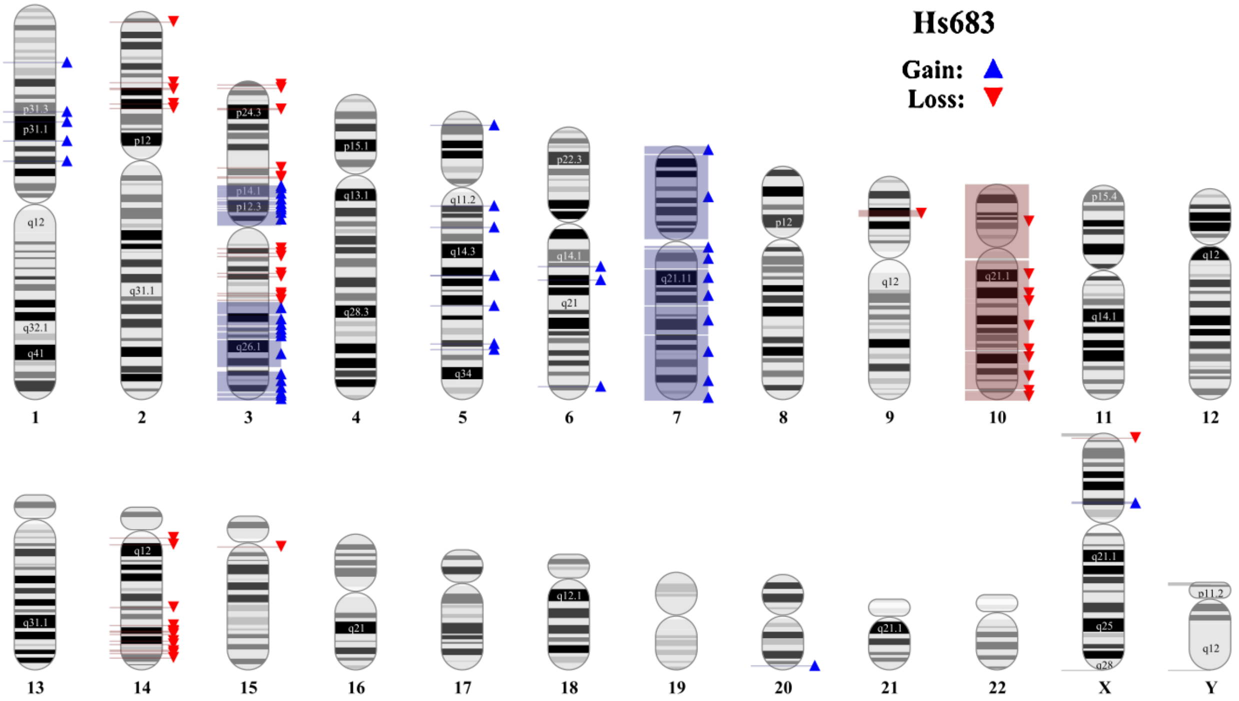

Supplementary Figure 1A DNA Mapping of Hs683 cells

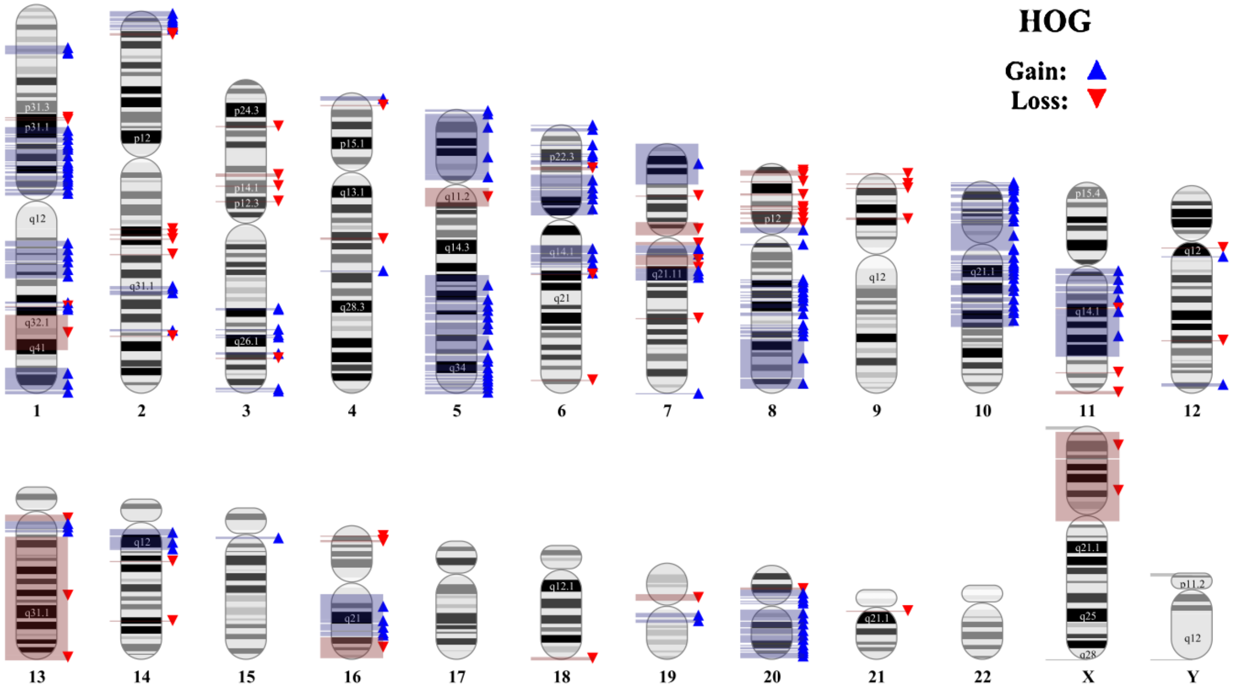

Supplementary Figure 1B DNA Mapping of HOG Cells

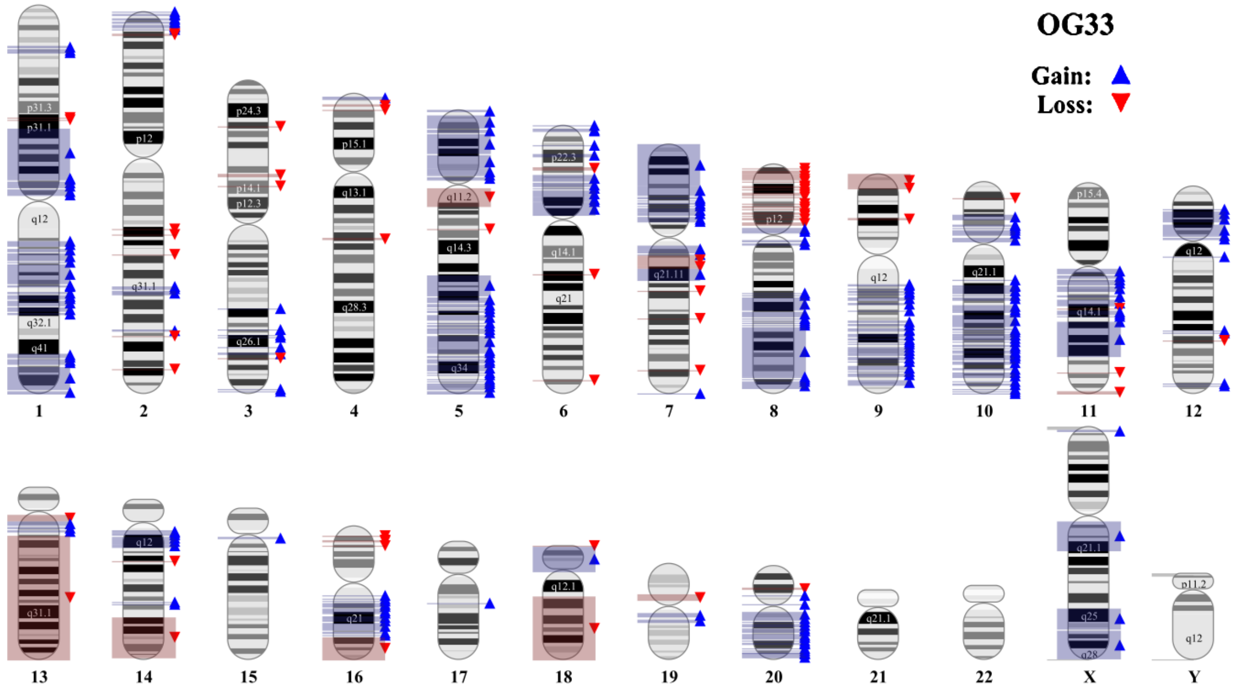

Supplementary Figure 1C DNA Mapping of OG33 cells

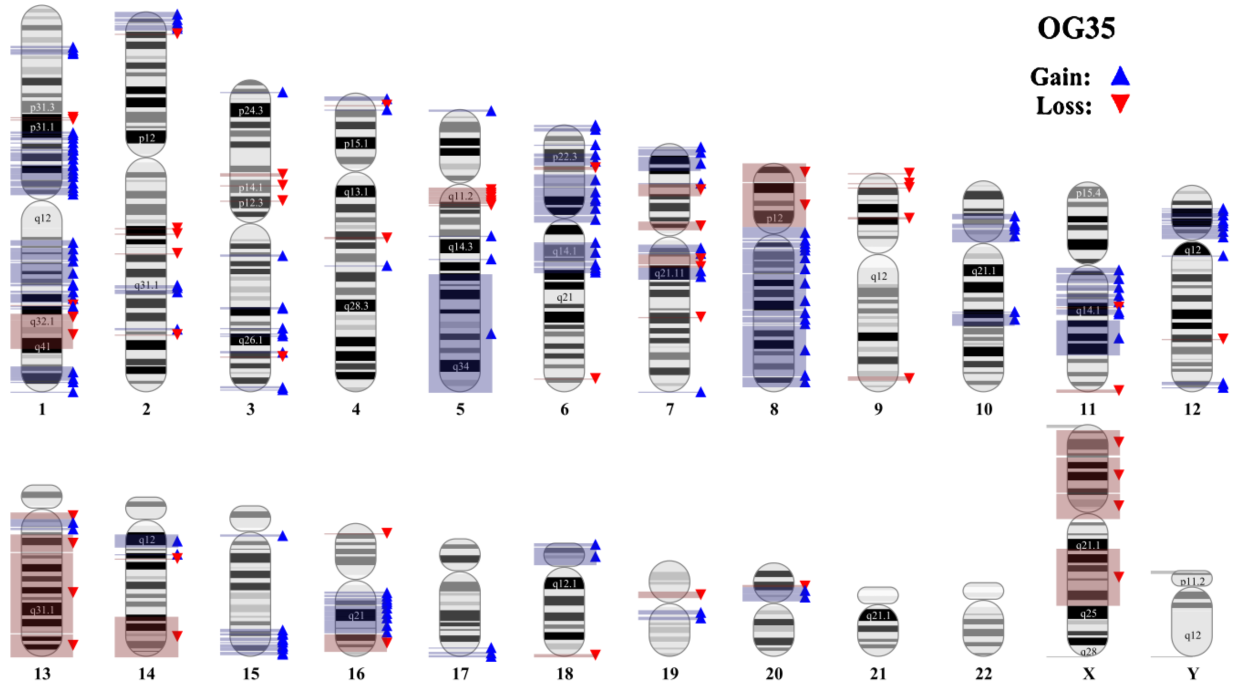

Supplementary 1D DNA Mapping of OG35 cells
