# Supplementary figures and images for: Acetate Supplementation Induces Growth Arrest of NG2/PDGFRα-Positive Oligodendroglioma-Derived Tumor-Initiating Cells
Source: PLoS One. 2013 Nov 20;8(11):e80714. doi: 10.1371/journal.pone.0080714 (PMC3835562; doi:10.1371/journal.pone.0080714)

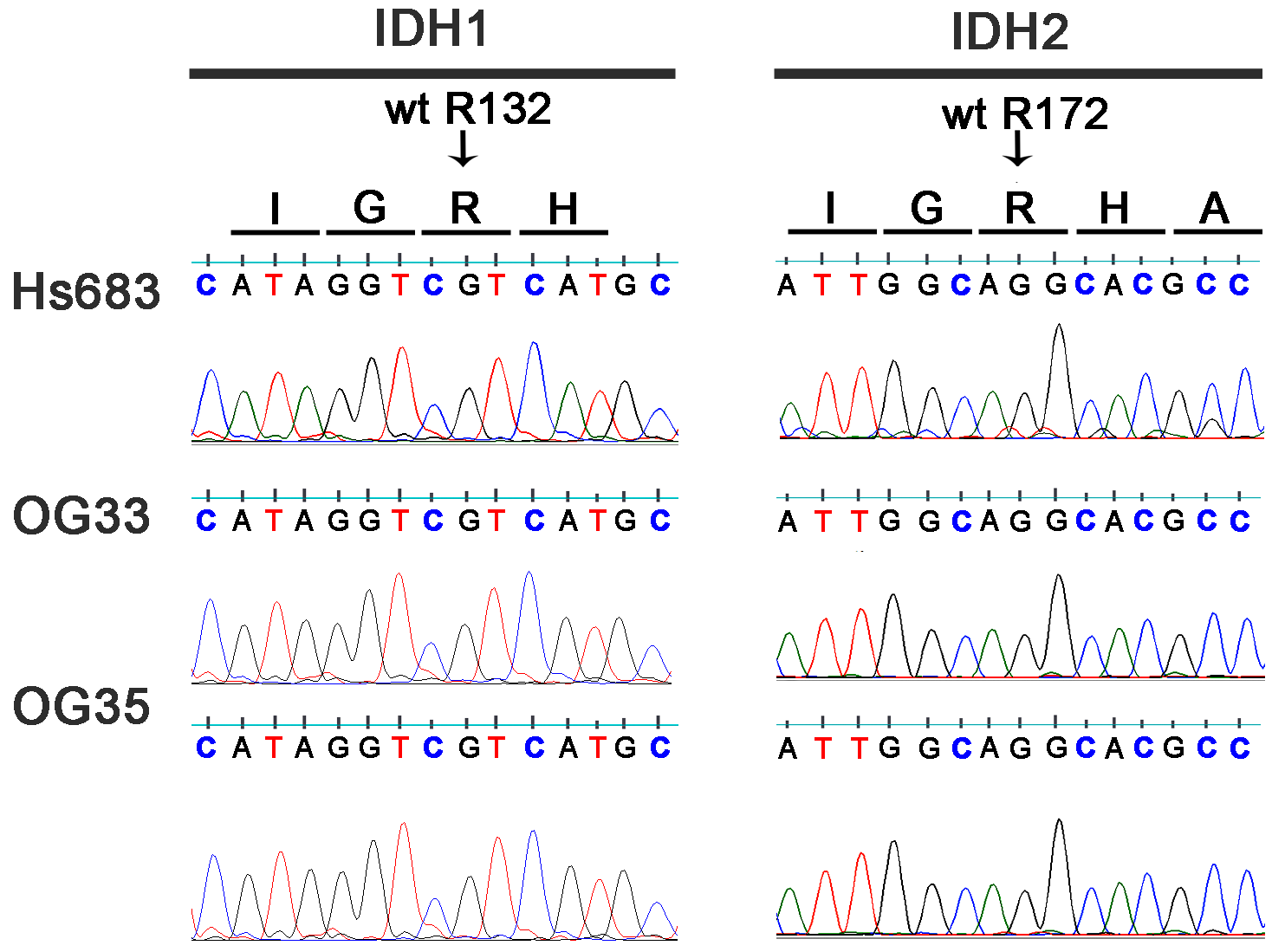

Supplement: Figure S2 — Hs683, OG33, and OG35 cells express wild-type IDH1 and IDH2. PCR amplification and sequencing of genomic DNA corresponding to exon 4 of IDH1 and IDH2 was performed to determine whether the cells harbored mutations in IDH1 (R132H: CAT, R132C: TGT, R132G: GGT, or R132S: AGT) or IDH2 (R172G: GGG, R172K: AAG, R172W: TGG or R172M: ATG). All cell lines possessed wild-type IDH1 (R132: CGT) and IDH2 (R172: AGG) sequences. (TIF) [file pone.0080714.s004.tif]

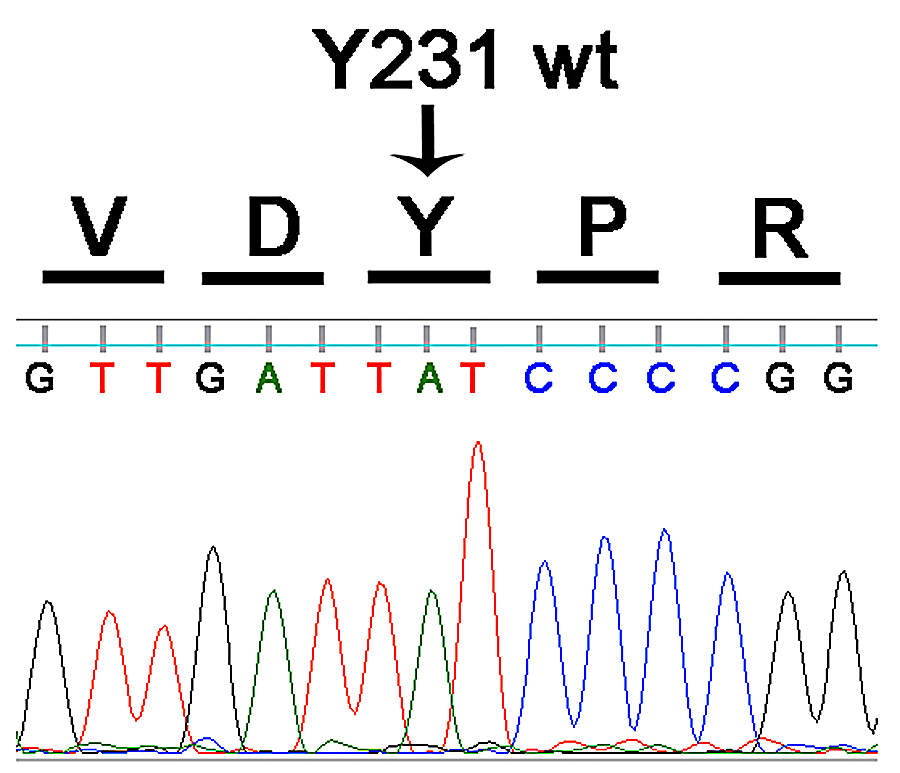

Supplement: Figure S3 — GTA alters morphology of oligodendroglioma cells and OPCs. Cells were plated in GM for 24 hours prior to treatment with 0.25% GTA for 1, 3, or 5 days (i.e., 2, 4, and 6 days in vitro). In addition to its stereotypical cytosolic localization, abundant ASPA immunoreactivity was present within the nucleus of all three cell lines. GTA induced cytosolic accumulation of ASPA and a profound morphological alteration in Hs683 cells, decreased ASPA immunoreactivity in HOG cells, and increased cytosolic ASPA accumulation in Oli-Neu processes. Similar to ASPA, AceCS1 was abundant in the nucleus and increased with time in culture in all three cell lines. CNPase immunoreactivity was modestly increased with time in culture in Hs683and HOG cells and GTA reduced labeling. Oli-Neu cells with a branched, differentiated morphology increased with time in culture even in SATO GM. GTA reduced the frequency of differentiated CNPase-positive Oli-Neu cells. Scale bar = 100 µm. (TIF) [file pone.0080714.s005.tif]

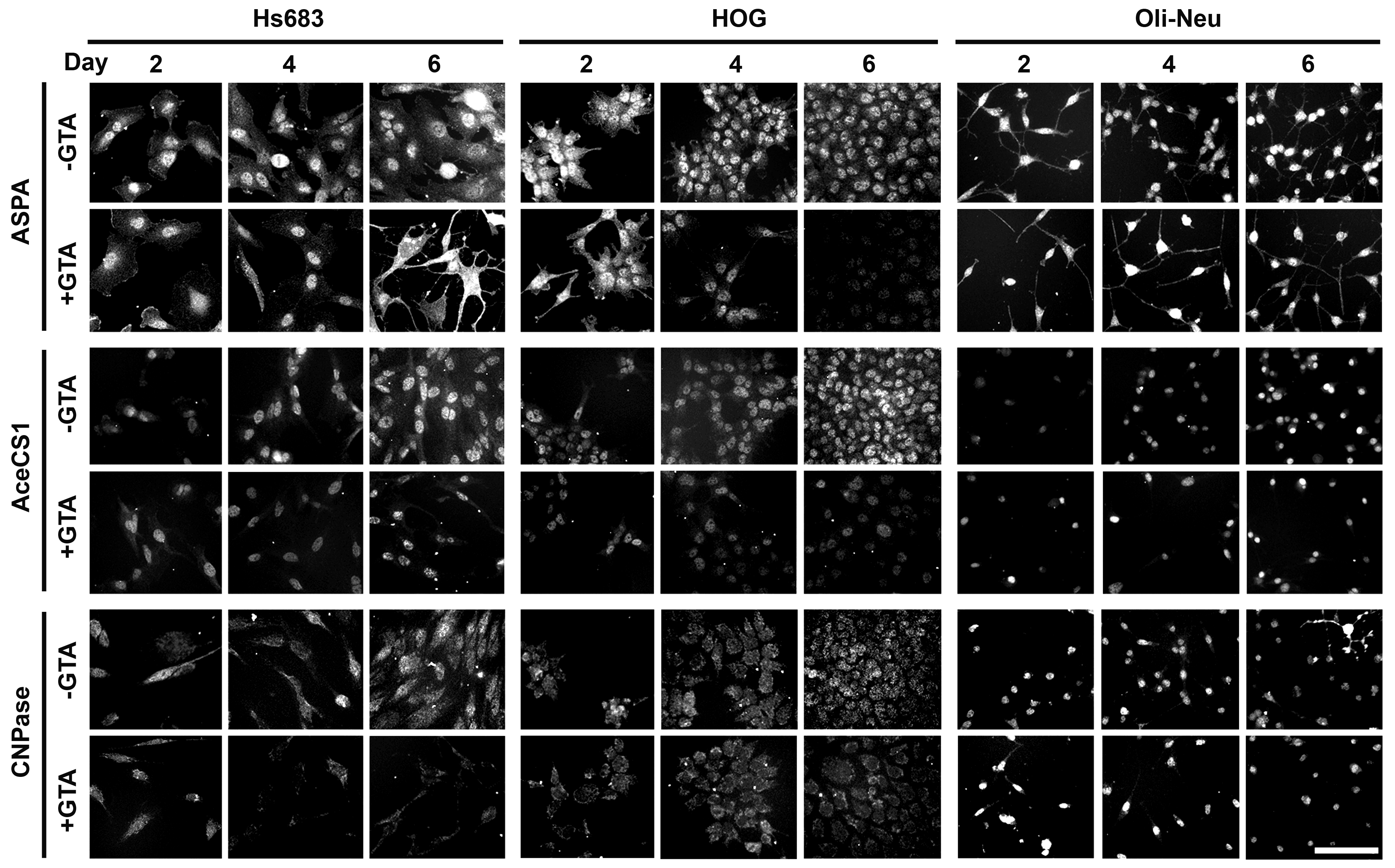

Supplement: Figure S4 — GTA mediated growth arrest is greater than the combination of 36 mM sodium acetate and 0.25% glycerol. OG33 and OG35 cells were plated in stem cell medium (SCM) as floating spheres or in differentiation medium (DM) in the absence or presence of 0.25% GTA, 36 mM sodium acetate (NaAc, equivalent acetate to 0.25% GTA), or 36 mM sodium acetate plus 0.25% glycerol (equivalent to what is derived from 0.25% GTA). Because GTA treatment in DM resulted in medium acidification of OG33 and, to a greater extent, OG35 cells between days 3 and 5, cells were treated on day 3 with acidified medium (pH 6.5, the maximum attained in OG35 GTA treated cells). Medium was replenished every 48 hours. Cell growth was determined after 1, 3, and 5 days of treatment by unbiased trypan blue based cytometry. Overall, 0.25% GTA more effectively reduced cell growth than 36 mM sodium acetate, with OG35 cells in SCM being the notable exception. Cell growth was comparable with 36 mM sodium acetate alone and the addition of 0.25% glycerol. The addition of acidified medium at day 3 did not alter cell growth. (TIF) [file pone.0080714.s006.tif]

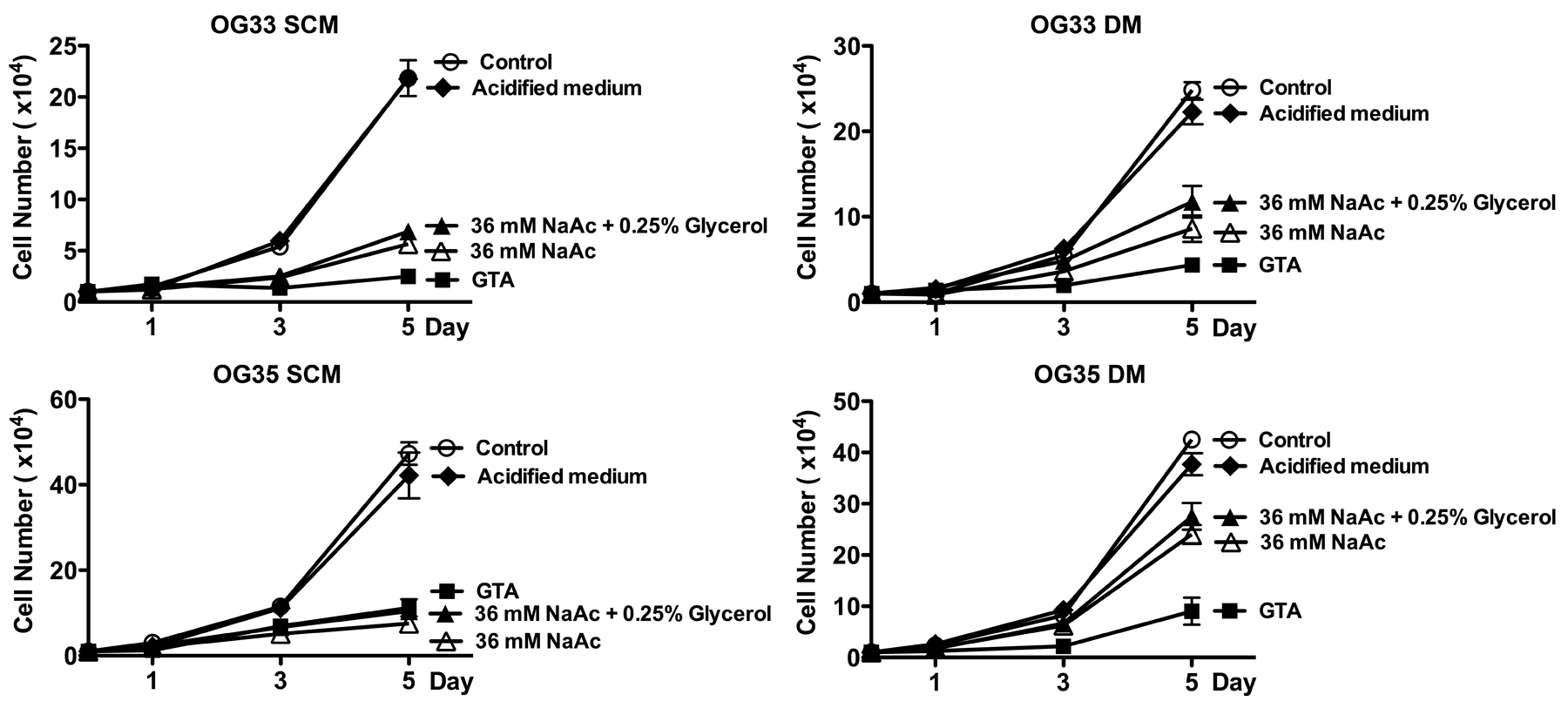

Supplement: Figure S5 — GTA does not reduce cell growth via apoptosis. Cells were grown in the absence or presence of 0.25% GTA in DM for 5 days and proliferation (phospho-Histone H3 [Ser10]), apoptosis (cleaved Poly ADP ribose polymerase, Asp214]), and cytoskeletal architecture (α-tubulin) were examined with the PathScan multiple immunofluorescence kit. GTA induced cytostasis via reduced proliferation, but not increased apoptosis. Scale bar = 100 μm. (TIF) [file pone.0080714.s007.tif]

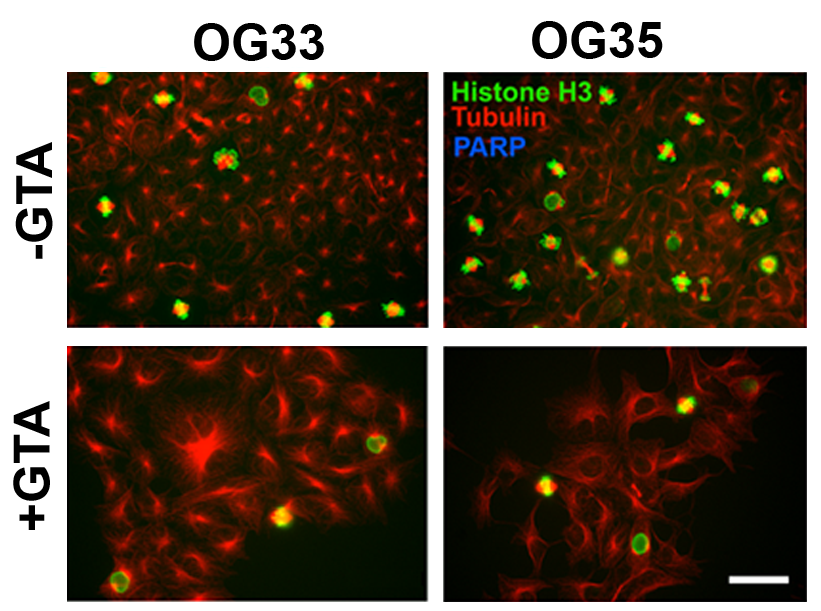

Supplement: Figure S6 — OG33 cells express wild-type ASPA. PCR amplification and sequencing of genomic DNA corresponding to exon 5 of ASPA was performed to determine whether the novel 26 kDa ASPA immunoreactive species present in OG33 cells (Figure 4) could arise from the most common ASPA mutation (i.e., Y231X), which results in a premature termination and a 26 kDa inactive protein. Codon 231 possessed a silent single nucleotide polymorphism (C/T) that would not affect protein coding. Thus, the 26 kDa protein in OG33 cells does not originate from premature termination within exon 5. (TIF) [file pone.0080714.s008.tif]

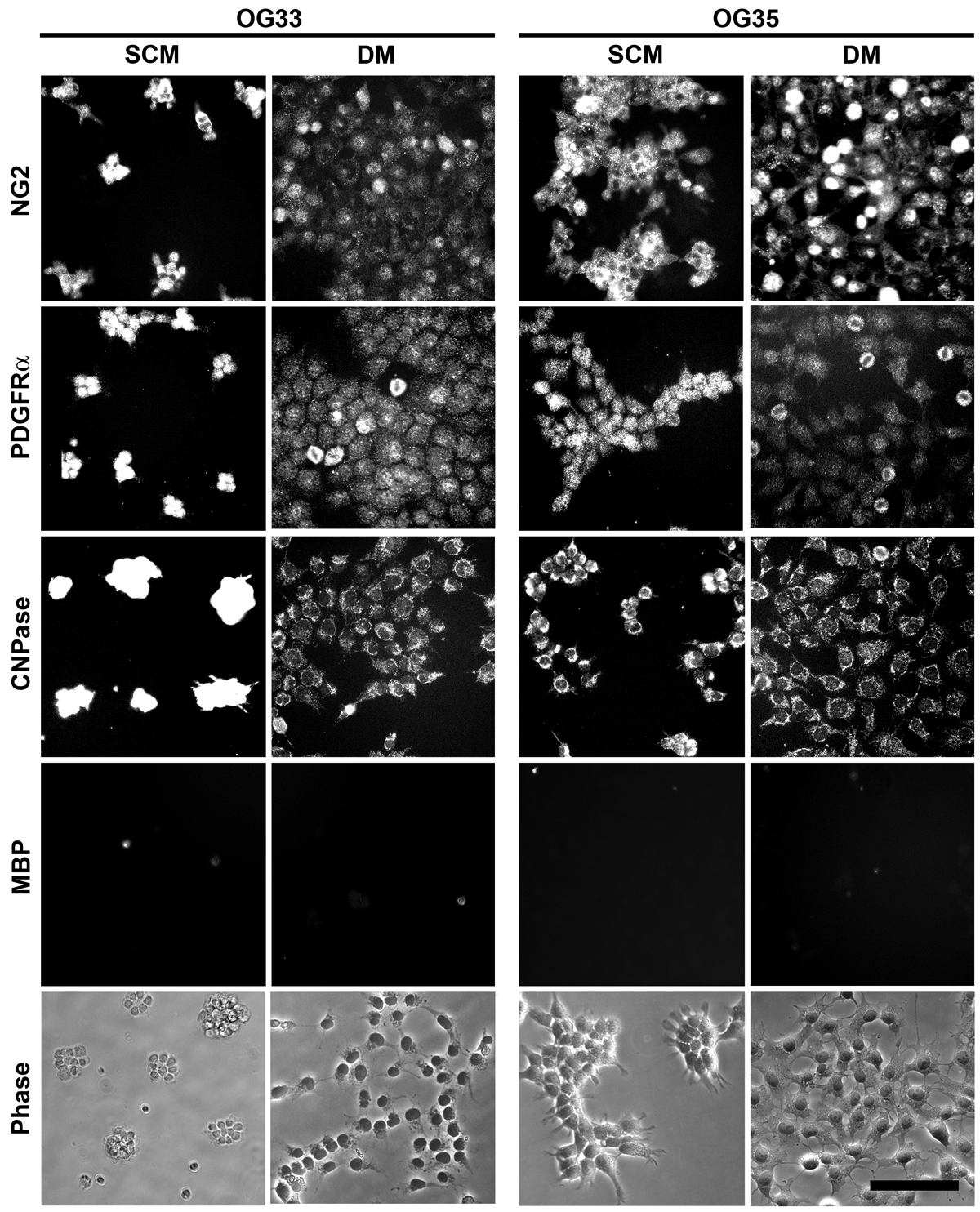

Supplement: Figure S7 — OG cells express markers of immature and young oligodendrocytes, but not myelin basic protein (MBP), a marker or more mature oligodendrocytes. OG33 and OG35 cells were plated in stem cell medium (SCM) adherently on PLL-coated wells or differentiation medium (DM) for 5 days. Both cells abundantly express NG2, PDGFRα, and CNPase in SCM and retain expression in DM; however, cells failed to express detectable MBP immunoreactivity. Scale bar = 100 μm. (TIF) [file pone.0080714.s009.tif]

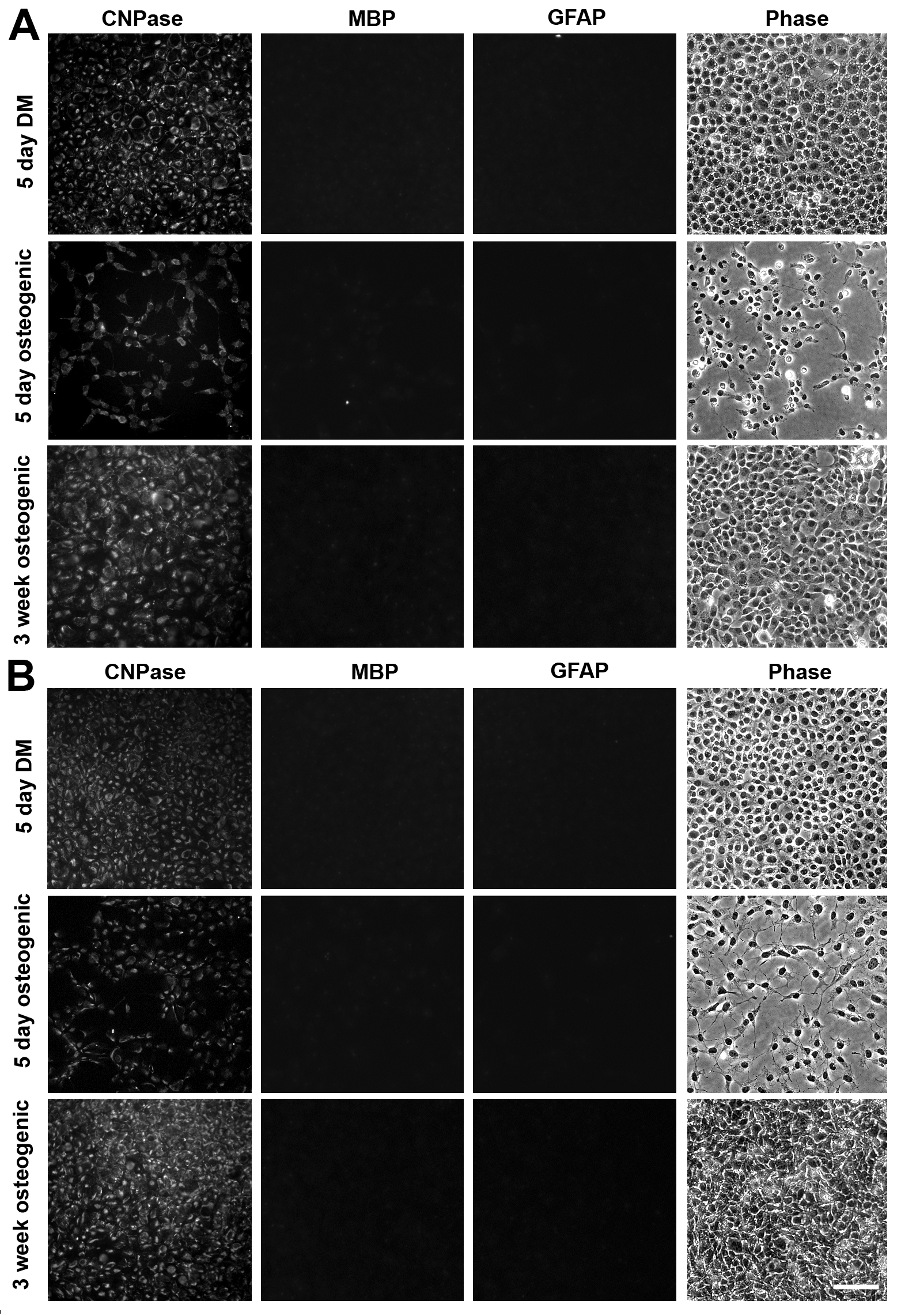

Supplement: Figure S8 — OG cells do not undergo osteoblastic differentiation. OG33 (A) and OG35 (B) cells were plated in DM (DMEM with 10% FBS) or osteogenic medium for up to 3 weeks. Medium was replenished every 3-4 days. After 5 days in osteogenic medium, reduced cell growth and altered cell morphology was apparent relative to cells in DM. However, in 3 week cultures, no Alizarin Red S positive cells or mineralized foci were detected (not shown). Furthermore, no increased CNPase immunoreactivity was observed and cells did not display immunoreactivity for myelin basic protein (MBP) or glial fibrillary acidic protein (GFAP). Thus, even protracted growth under differentiation conditions failed to induce mature glial or mesenchymal differentiation. Scale bar = 100 μm. (TIF) [file pone.0080714.s010.tif]
